# Usability Assessment Methods for Mobile Apps for Physical Rehabilitation: Umbrella Review

**DOI:** 10.2196/49449

**Published:** 2024-10-04

**Authors:** Sylvia Hach, Gemma Alder, Verna Stavric, Denise Taylor, Nada Signal

**Affiliations:** 1 Health and Rehabilitation Research Institute Faculty of Health and Environmental Sciences Auckland University of Technology Auckland New Zealand

**Keywords:** usability, quality evaluation, mobile health, physical exercise, rehabilitation, overview, umbrella review, psychometrics

## Abstract

**Background:**

Usability has been touted as one determiner of success of mobile health (mHealth) interventions. Multiple systematic reviews of usability assessment approaches for different mHealth solutions for physical rehabilitation are available. However, there is a lack of synthesis in this portion of the literature, which results in clinicians and developers devoting a significant amount of time and effort in analyzing and summarizing a large body of systematic reviews.

**Objective:**

This study aims to summarize systematic reviews examining usability assessment instruments, or measurements tools, in mHealth interventions including physical rehabilitation.

**Methods:**

An umbrella review was conducted according to a published registered protocol. A topic-based search of PubMed, Cochrane, IEEE Xplore, Epistemonikos, Web of Science, and CINAHL Complete was conducted from January 2015 to April 2023 for systematic reviews investigating usability assessment instruments in mHealth interventions including physical exercise rehabilitation. Eligibility screening included date, language, participant, and article type. Data extraction and assessment of the methodological quality (AMSTAR 2 [A Measurement Tool to Assess Systematic Reviews 2]) was completed and tabulated for synthesis.

**Results:**

A total of 12 systematic reviews were included, of which 3 (25%) did not refer to any theoretical usability framework and the remaining (n=9, 75%) most commonly referenced the ISO framework. The sample referenced a total of 32 usability assessment instruments and 66 custom-made, as well as hybrid, instruments. Information on psychometric properties was included for 9 (28%) instruments with satisfactory internal consistency and structural validity. A lack of reliability, responsiveness, and cross-cultural validity data was found. The methodological quality of the systematic reviews was limited, with 8 (67%) studies displaying 2 or more critical weaknesses.

**Conclusions:**

There is significant diversity in the usability assessment of mHealth for rehabilitation, and a link to theoretical models is often lacking. There is widespread use of custom-made instruments, and preexisting instruments often do not display sufficient psychometric strength. As a result, existing mHealth usability evaluations are difficult to compare. It is proposed that multimethod usability assessment is used and that, in the selection of usability assessment instruments, there is a focus on explicit reference to their theoretical underpinning and acceptable psychometric properties. This could be facilitated by a closer collaboration between researchers, developers, and clinicians throughout the phases of mHealth tool development.

**Trial Registration:**

PROSPERO CRD42022338785; https://www.crd.york.ac.uk/prospero/#recordDetails

## Introduction

The development of mobile health (mHealth) [1,2] solutions has seen exponential growth in recent times, driven particularly by the global pandemic [3,4]. mHealth has been heralded as a tool to provide access to quality rehabilitation input for patients outside of the time they are able to spend with clinicians [5] and for patients in geographically remote areas [6]. Furthermore, similar to the observed trend of increased health information seeking on the internet [7], the democratization of access to rehabilitation could be achieved by individuals actively seeking stand-alone mHealth solutions.

However, there is also increasing awareness that mHealth solutions available to clinicians and their patients often lack quality evaluations [8,9]. Many mHealth solutions only have short-term (<30 days) data from small sample sizes to support their effectiveness [10]. Moreover, only limited standardized outcome measures are typically used [11,12].

Usability is one key aspect commonly included in the evaluation of mHealth solutions [5,9,11]. It has been touted as a determiner of the success of mHealth interventions [13]. Usability is often delineated from two related concepts: (1) the concept of utility that captures a system’s ability to meet user needs [14] and (2) user experience is commonly understood as a broader concept of the experience of using an mHealth solution and may include measures of user beliefs [15]. However, usability may or may not be part of how user experience is captured, and many different definitions of usability appear in the literature [15-17].

The diversity in definitions of usability is mirrored by the diversity in usability models or frameworks. The 5 most commonly cited models of usability are that of ISO9241-11 [18] and its revision [19]; ISO/IEC25010 [20]; Nielsen’s usability model [21]; and, in the context of health in particular, the People At the Centre of Mobile Application Development (PACMAD) model [14,22]. These models identify factors such as efficiency, or the resources expended to achieve a task; effectiveness, the level of accuracy and completeness of a task achieved using a mobile solution; and satisfaction or positive user interaction while operating the mobile solution as components of usability. The key difference between the PACMAD and the aforementioned frameworks is that these and other factors such as errors are seen as arising from 3 different sources: the user themselves, the task, and the context. This could be argued to be of particular importance for mHealth, where users may experience limitations such as perceptual or cognitive (aging) barriers [23]. These additionally impact on task demands and therefore represent an important consideration in the design of mHealth tools.

Usability assessment has been included in several good practice guidelines for the development of mHealth solutions [24-28], as well as in many evaluation frameworks [29,30], and can be regarded as a crucial step for evaluation at different stages of the typical mHealth development cycles. To date, however, no accepted standard for the assessment of usability of mHealth solutions exists. This means that researchers and developers of mHealth are faced with difficult decisions when designing mHealth evaluation procedures that strike the balance between responsiveness, reliability, and validity and are unable to compare existing solutions for the purpose of innovating. Further, clinicians are unable to be guided in their prescription of mHealth solutions, and there are significant barriers for consumers to engage with existing solutions.

Numerous systematic reviews have explored usability assessment approaches for various mHealth solutions in the context of physical rehabilitation. However, there is a lack of synthesis in this area of the literature. This may contribute to clinicians and developers needing to devote a significant amount of time and effort in analyzing and summarizing a large body of systematic reviews. An umbrella review can act as “a means for a rapid review of the evidence to address a broad and high-quality evidence base” [31]. Specifically, an umbrella review allows for a broader scope than individual systematic reviews that may focus on individual treatment options or individual conditions [32-34]. Hence, the aim of this umbrella review was to provide a “user-friendly” summary of the use of usability assessment instruments, or measurement tools, for researchers, clinicians, and consumers of mHealth irrespective of the specific area of application (eg, diabetes, tuberculosis, and sleep). Specifically, the objective was to summarize systematic reviews that investigated usability assessment instruments in mHealth interventions including those related to physical exercise rehabilitation. It is envisaged that such a summary will first aid researchers, developers, and clinicians to gain an overview of usability assessment instruments without needing to explore primary literature. Second, the presented summary may aide the development of mHealth usability assessment standards.

## Methods

### Overview

The umbrella review protocol was developed based on the *Cochrane Handbook for Systematic Reviews of Interventions* [33] and other relevant methodology sources [34] and was registered with PROSPERO (CRD42022338785). StArt (State of the Art through Systematic Review) software [35] was used for the first- and second-level screening of result datasets and extracting relevant information.

### Inclusion Criteria

Based on the objectives of the study, the following inclusion criteria were formulated: (1) articles published between January 1, 2015, and April 27, 2023 (the date range reflected the launch of Apple ResearchKit in 2015, which accelerated mHealth development and research [36]); (2) containing data on human participants; (3) with the “unit of searching” [33] being “systematic reviews” [37,38] in order to reduce the effect of cumulative bias that may arise when including nonsystematic reviews; (4) examining usability assessment instruments of mobile apps for health professionals and for health care consumers; and (5) published in the English language to enable all contributing authors to perform screening, extraction, and synthesis of the search results. No post hoc modifications were made to the inclusion criteria. Systematic reviews of usability assessment instruments of other (mobile) solutions such as wearables, sensors, virtual reality, blockchain, Internet of Things, simulated data, or solutions for health care professionals only were excluded.

### Search Methods and Search Terms

The following databases were searched with a combination of the search terms mobile application*, mobile app, usab*, usab* criteria, usab* evaluat*, systematic review, mhealth, mobile health, and physical exercise: PubMed, Cochrane, IEEE Xplore, Epistemonikos, Web of Science, and CINAHL Complete, combined using Boolean operators OR and AND and customized for each database in accordance with their filtering specifications. The result sets were imported into StArt [35]. The full search syntax for each database are presented in Table S1 in Multimedia Appendix 1.

### Data Collection and Analysis

A preliminary search of existing systematic reviews was conducted before finalizing the search terms in order to scope the extent and type of existing evidence [33]. The subsequent final search terms produced a result set that was more refined in focus and feasible in terms of the size of the expected result set. Following the removal of duplicates, 2-level screening was performed: title and abstract screening was performed by the primary author (SH), and a randomly selected subset of articles (118/1479, approximately 8%) was screened by a second author (VS; κ=0.87). Second-level, full-text screening was performed by the primary author (SH) using StArt for data extraction from the final result set. A data extraction form including basic reference details, as well as information such as population of interest and interventions studied, was discussed and agreed on by 3 authors (SH, GA, NS) before data extraction (see review protocol PROSPERO CRD42022338785 for more detail).

Quality assessment was completed using AMSTAR 2 (A Measurement Tool to Assess Systematic Reviews 2; Institute for Clinical Evaluative Sciences) [39] by the primary author (SH) and a second author (VS) separately (κ=0.823). Any disagreement was discussed and resolved via consensus. In line with recommendations by Shea et al [39], a discussion to determine AMSTAR 2 critical domains for this umbrella review occurred among 2 authors (SH, NS). Criteria 2, 4, and 7 were retained on the premise of constituting critical criteria as defined by the original publication [39]. The original critical criteria 9, 11, 13, and 15 were classified as noncritical for the purpose of this umbrella review due to pertaining to meta-analytic steps that none of the included systematic reviews performed. Instead, the following criteria were classified as critical: criterion 5 due to the variety of study designs and target user groups and/or clinical contexts included within the systematic reviews; and criterion 16 due to the context of mHealth usability, where the borders between academic enquiry and commercialization are more blurred and funding could constitute a significant source of bias and/or conflict of interest. A summary rating was produced according to recommendations by Shea et al [39].

Finally, to gauge potential skewing of the data caused by significant overlap of primary studies contained within the systematic reviews included in this umbrella review [40], overlap assessment was achieved via citation matrix [41,42] for the systematic reviews including the System Usability Scale (SUS) as an exemplar. The SUS was chosen because it is one of the most well-known instruments [43] and preliminary searches of the literature demonstrated its frequency of use and reference.

## Results

**Figure 1:**
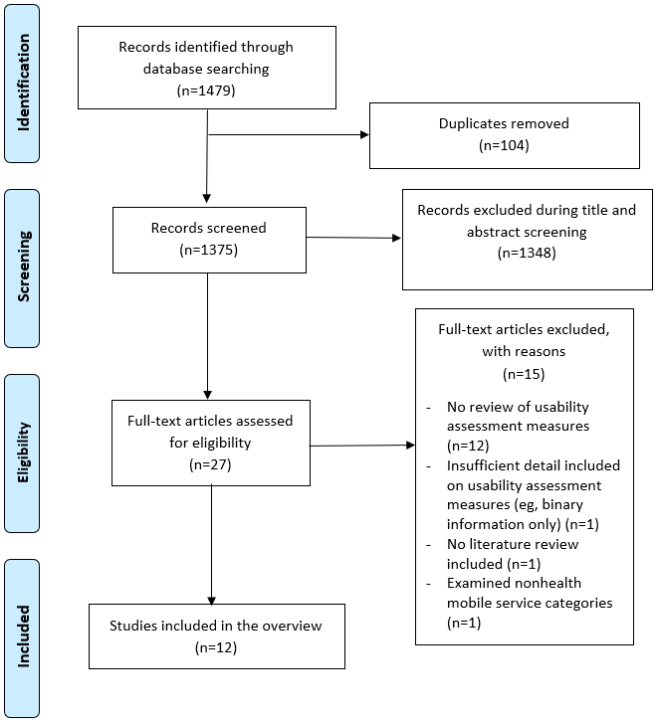
PRISMA (Preferred Reporting Items for Systematic Reviews and Meta-Analyses) flowchart.

The initial database search returned 1479 results, which were reduced to 1375 after removal of duplicates (see Figure 1). Title and abstract screening resulted in 27 articles being included for full-text screening. A total of 15 of the full-text articles retrieved (see Table S2 in Multimedia Appendix 1) were ineligible because they did not review usability assessment measures, include sufficient detail on usability assessment instruments (eg, including binary information only), include a literature review, or examine nonhealth mobile service categories (see Figure 1).

A total of 12 systematic reviews examining usability assessment instruments were included. Data were extracted (see Table S3 in Multimedia Appendix 1) as per the registered protocol. Across the systematic reviews included, there was coverage of primary studies from the start of records to 2020. Three of the systematic reviews included examined usability assessment instruments within a specific target user group (eg, users with diabetes [44] and users living with a mental health concern [45,46]). The remaining 9 systematic reviews [13,47-54] focused on usability assessment instruments used across different target user populations. Usability models or frameworks referenced included ISO [20] (referenced in [13,44,48,49]), Nielsen [21] (referenced in [45]), and the framework by the Canadian Institutes of Health Research and the Mental Health Commission Canada [55] (referenced in [47]). Three (25%) of the systematic reviews [46,50,51] included in this umbrella review did not refer to any theoretical framework (see Table S3 in Multimedia Appendix 1).

The systematic reviews included identified a total of 32 usability assessment instruments (see Table 1) and a further 66 custom-made usability assessment instruments as well as hybrid custom-made instruments (see Table S4 in Multimedia Appendix 1). The most commonly referenced usability assessment instrument was the SUS [56], followed by the IBM Computer Usability Satisfaction Questionnaire [57] and the Usefulness, Satisfaction, and Ease of Use (USE) Questionnaire [58].

**Table 1:** Overview of usability assessment scales identified by reviews included within this umbrella review.

Assessment scale	Reference	Systematic review identifying scale	Count	Psychometric properties as identified by systematic reviews included in this umbrella review						
				Internal consistency (Cronbach α)	Reliability (intraclass correlation)	Content validity	Structural validity	Cross-cultural validity	Criterion, convergent, concurrent, discriminant validity	Responsiveness
App adaptation Abbott’s scale	[59]	Nouri et al [50]	1	NR^a^	NR	NR	NR	NR	NR	NR
After Scenario Questionnaire	[60]	Inal et al [45]	1	NR	NR	NR	NR	NR	NR	NR
App adaptation Brief DISCERN	[59]	Nouri et al [50]	1	NR	NR	NR	NR	NR	NR	NR
App adaptation CRAAP checklist	[61]	Nouri et al [50]	1	NR	NR	NR	NR	NR	NR	NR
Ease of Use and Usefulness Scale (EUUS)	[62]	Kien et al [53]	1	NR	NR	NR	NR	NR	NR	NR
Enlight	[63]	Azad-Khaneghah et al [47]	1	NR	NR	NR	NR	NR	NR	NR
Health Information Technology Usability Evaluation Scale (Health-ITUES)	[64]	Azad-Khaneghah et al [47], Muro-Culebras et al [51]	2	0.85-0.92	No	Expert panel and factor analysis	Exploratory and confirmatory factor analysis	No	Correlation with the Post-Study System Usability Questionnaire (PSSUQ)	Statistically significant difference was demonstrated with the intervention group
Health IT Usability Evaluation Model (Health-ITUEM)	[65]	Nouri et al [50], Vera et al [48]	2	NR	NR	NR	NR	NR	NR	NR
App adaptation Health-Related Website Evaluation Form (HRWEF)	[66]	Nouri et al [50]	1	NR	NR	NR	NR	NR	NR	NR
App adaptation Health On the Net (HON) code	[59]	Nouri et al [50]	1	NR	NR	NR	NR	NR	NR	NR
IBM Computer Usability Satisfaction Questionnaire	[57]	Azad-Khaneghah et al [47], Georgsson [44], Ng et al [46], Wakefield et al [52], Zapata et al [13]	5	0.89	No	Expert panel	No	NR	No	No
ISOMETRIC	[67]	Azad-Khaneghah et al [47]	1	NR	NR	NR	NR	NR	NR	NR
iSYScore index	[68]	Muro-Culebras et al [51]	1	No	No	Expert panel	No	NR	No	No
App adaptation Kim Model	[69]	Nouri et al [50]	1	NR	NR	NR	NR	NR	NR	NR
Measurement Scales for Perceived Usefulness and Perceived Ease of Use	[70]	Muro-Culebras et al [51]	1	0.97 (usefulness), 0.91 (ease of use)	No	Focus group	Exploratory factor analysis	No	Convergent and discriminant validity	No
Mobile App Rating Scale (MARS)	[71]	Muro-Culebras et al [51], Nouri et al [50], Vera et al [48]	3	0.90	0.79	Expert panel	No	No	No	No
Mobile App Rating Scale (user version) (uMARS)	[72]	Muro-Culebras et al [51], Nouri et al [50]	2	0.90	0.66 (1-2 mo), 0.70 (3 mo)	Expert panel and focus groups	No	No	No	No
NASA Task Load Index (TLX)	[73]	Zapata et al [13]	1	NR	NR	NR	NR	NR	NR	NR
NICE guidelines tool	[74]	Azad-Khaneghah et al [47]	1	NR	NR	NR	NR	NR	NR	NR
Perceived Useful and Ease of Use Questionnaire (PUEU)	[75]	Azad-Khaneghah et al [47], Inal et al [45]	2	NR	NR	NR	NR	NR	NR	NR
Post-Study System Usability Scale (PSSUS)/PSSUQ	[76]	Inal et al [45], Niknejad et al [54], Vera et al [48]	3	NR	NR	NR	NR	NR	NR	NR
Quality Assessment tool for Evaluating Medical Apps (QAEM)	[77]	Azad-Khaneghah et al [47]	1	NR	NR	NR	NR	NR	NR	NR
Quality of Experience (QOE)	[78]	Azad-Khaneghah et al [47], Nouri et al [50]	2	NR	NR	NR	NR	NR	NR	NR
Questionnaire for User Interaction Satisfaction 7.0 (QUIS)	[79]	Georgsson [44], Saeed et al [49]	2	NR	NR	NR	NR	NR	NR	NR
App adaptation Silberg score	[80]	Azad-Khaneghah et al [47], Nouri et al [50]	2	NR	NR	NR	NR	NR	NR	NR
Software Usability Measurement Inventory (SUMI)	[81]	Azad-Khaneghah et al [47]	1	NR	NR	NR	NR	NR	NR	NR
System Usability Scale (SUS)	[56]	Azad-Khaneghah et al [47], Georgsson [44], Inal et al [45], Muro-Culebras et al [51], Ng et al [46], Niknejad et al [54], Nouri et al [50], Vera et al [48], Wakefield et al [52], Zapata et al [13]	10	0.911	No	Focus group	Exploratory and confirmatory factor analysis	No	No	No
Telehealth Usability Questionnaire (TUQ)	[82]	Georgsson [44], Inal et al [45], Niknejad et al [54]	3	NR	NR	NR	NR	NR	NR	NR
Telemedicine Satisfaction and Usefulness Questionnaire (TSUQ)	[83]	Wakefield et al [52]	1	0.96 (video visits), 0.92 (use and impact)	No	Expert panel	Exploratory factor analysis	No	Significant discriminant validity (Hispanic vs non-Hispanic)	No
The mHealth App Usability Questionnaire for interactive mHealth apps (patient version) (MAUQ)	[84]	Muro-Culebras et al [51]	1	0.895, 0.829, 0.900	No	Expert panel	Exploratory factor analysis	No	Correlation with PSSUQ and SUS	No
The mHealth App Usability Questionnaire for standalone mHealth apps (patient version) (MAUQ)	[84]	Muro-Culebras et al [51]	1	0.847, 0.908, 0.717	No	Expert panel	Exploratory factor analysis	No	Correlation with PSSUQ and SUS	No
Usefulness, Satisfaction, and Ease of Use (USE) Questionnaire	[58]	Azad-Khaneghah et al [47], Inal et al [45], Kien et al [53], Ng et al [46]	4	NR	NR	NR	NR	NR	NR	NR

^a^NR: not reported as part of the systematic reviews included in this umbrella review.

Data regarding the psychometric properties of 9 (28%) instruments [56,57,64,70-72,83,84] were included in the systematic reviews as detailed in Table 1. Internal consistency was generally good across these instruments, content validity was provided through expert panel or focus groups [56,57,64,70,71,83,84], and exploratory and/or confirmatory factor analyses were used in evidence of structural validity [56,64,70,83,84]. Details of convergent validity were included for 3 instruments [64,70,84] (see Table 1). Importantly, there was no evidence of reliability, responsiveness, or cross-cultural validity assessment for the usability assessment instruments referenced most often (ie, SUS, IBM Computer Usability Satisfaction Questionnaire, and USE Questionnaire).

Further, 8 (67%) of the systematic reviews [13,44-46,48-50,54] referred to usability assessment methods other than assessment scales. These included focus groups, heuristic evaluation, think-aloud protocols, and other methods (see Table S5 in Multimedia Appendix 1).

**Figure 2:**
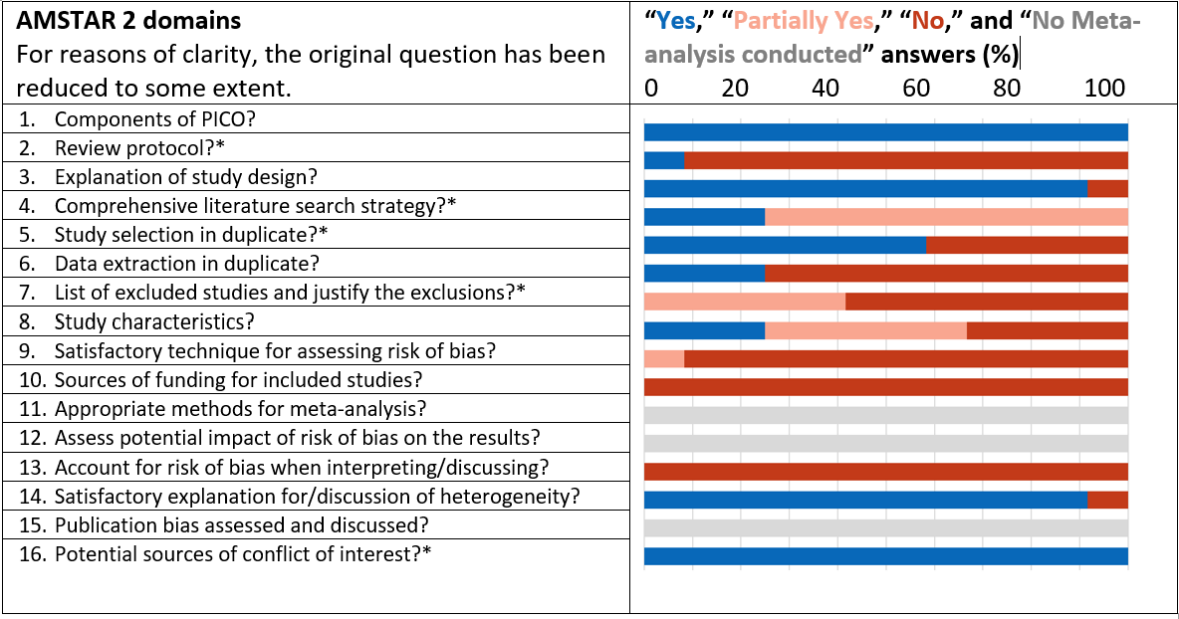
Overview of methodological quality of reviews according to AMSTAR 2 (A Measurement Tool to Assess Systematic Reviews 2). * denotes critical criterion as determined for this umbrella review.

Quality assessment of the systematic reviews using AMSTAR 2 revealed that 8 (67%) articles [13,44-46,48-50,54] exhibited at least 2 critical weaknesses (see Figure 2), 3 (25%) systematic reviews [47,51,52] were affected by 1 critical weakness, and 1 (8%) review [53] had only noncritical weaknesses. The most frequently unfulfilled assessment criteria included the sources of funding enquiry for the included studies (AMSTAR criterion 10), accounting for risk of bias when interpreting results (AMSTAR criterion 13), use of a satisfactory technique for assessing risk of bias (AMSTAR criterion 9), and inclusion of a review protocol (AMSTAR criterion 2; see Table S6 in Multimedia Appendix 1).

Finally, visualization of citation overlap for systematic reviews including primary studies using the SUS showed minimal overlap with 4 (10%) of 41 primary studies included in 2 of the systematic reviews (see Table S7 in Multimedia Appendix 1). With the exception of the citation of the original publication of the SUS instrument [56], all other references included in the overview were unique to one of the systematic reviews included.

## Discussion

### Principal Findings

The exponential growth of research evidence related to the effectiveness of mobile solutions for rehabilitation [85-88] and the proliferation of technological solutions that afford new modes of treatment delivery [89,90] underscore the critical need for high-quality mHealth usability evaluation. Usability attributes such as efficiency, learnability, and memorability [21] are particularly important to consider for mHealth users who may face challenges due to neurological compromise [91], age-related issues [23], or limited technology experience [13]. This umbrella review aimed to summarize usability assessment instruments for mHealth researchers, clinicians, and consumers to guide the development, assessment, and selection of high-quality mHealth tools.

The review identified, first, significant diversity and common use of custom-made instruments when usability assessment instruments were employed to evaluate mHealth tools for rehabilitation. Second, there was a notable lack of theoretical grounding for selection of the assessment of usability. Third, a scarcity of psychometric data for widely used instruments for mHealth usability assessment was evident in the systematic reviews included.

### Heterogeneity of Instruments, Including Nonstandardized Instruments

Regarding the first critical point, a wide range of different instruments for the assessment of usability was evident across the systematic reviews included. This range included adaptations of preexisting usability assessment instruments for the context of mobile apps [59,66] as well as assessment instruments, such as the Mobile App Rating Scale (MARS) [72], specifically designed for usability assessment of mHealth tools. In addition, both completely custom-made instruments and hybrids [92] of preexisting instruments with custom elements were prevalent in the mHealth usability literature.

Although the use of hybrid assessment instruments and adaptations of preexisting assessment instruments may increase flexibility and thereby possibly improve the experience for respondents, the fact that most studies are limited in sample size prevents validation of hybrid and adapted instruments [51]. Alternative approaches to increasing flexibility and improving respondent experience while ensuring psychometric integrity are needed instead. A good example of this may be seen in the creation of a hybrid version of the SUS with the inclusion of pictorial elements, which increased respondent motivation [92]. Importantly, acceptable validity, consistency, and sensitivity were also evidenced, allowing future users of the hybrid measure to place greater trust in the quality of the data.

### Theoretical Underpinning

Second, and similar to what has been found to be the case for individual-level studies assessing the usability of specific mHealth tools [47], this review revealed that some systematic reviews examining the broader literature related to usability assessment lacked connection to theoretical models of usability. This observation resonates with previous criticisms of the quality of reviews of health-related mobile apps [8] as well as research exploring technology adoption in fields beyond mHealth [93]. The latter exposed a reliance on a wide array of theoretical models of technology adoption in the literature and in some cases several within one review. To address this, it has been suggested that generic models for different service categories (eg, information and transaction) be developed [93]. A theoretically grounded, generic guide for mHealth usability assessment could similarly promote broader adoption and enhance comparison of usability across studies and use cases.

### Psychometric Properties and Psychometric Testing

Third, systematic reviews included in our overview also reported significant limitations regarding the psychometric properties of preexisting instruments. For example, the MARS tool, which has been put forward as an instrument for standardized use in mHealth usability assessment [51], lacks structural validity. Moreover, other constructs such as internal consistency and criterion validity have been documented as significant areas of future work for measuring the implementation of interventions [53], with usability assessment playing a significant role.

Although consistent with previous research, this umbrella review did not specifically search for psychometric evaluations of usability assessment instruments; instead, it relied on summaries of psychometric evaluations presented as part of the included systematic reviews. As a result, it is likely that psychometric evaluation of other instruments is available. For example, psychometric evaluation of the popular USE Questionnaire [58] is available and, consistent with our observation, has been shown to be affected by a lack of reliability and validity [94]. Furthermore, outside of the academic literature, there is a still greater portion of mHealth solutions on the market that likely will not have undergone empirical evaluation of usability.

Although some of the acceptable psychometric information was referenced for the SUS [95], both the IBM Usability Satisfaction Questionnaire and the USE Questionnaire appear to lack reliability assessment. Reliability, or the freedom of measurement error [96], may be regarded as crucial with regard to any metrics that are gathered after, rather than during, a user’s interaction with an application. The inability to separate true change in users’ estimate of the usability of mHealth tools from random variation, or measurement error, originating from recall bias [9,97,98], for example, means that mHealth tool iterations [99] are unable to be evaluated appropriately.

Moreover, the widespread use of custom-made and hybrid assessment instruments leads to the loss of the original instrument’s integrity and compromises its already-documented psychometric strengths [100]. Consequently, establishing the validity of results from individual usability investigations becomes challenging, and comparison across studies is difficult. Hence, there is an urgent need to assess the accuracy and appropriateness [101] of individual usability assessment instruments to capitalize on the promise of mHealth tools in rehabilitation [5,6].

Another important psychometric aspect of usability assessment instruments that the systematic reviews included in this umbrella review highlight as missing from the published literature is responsiveness. mHealth development usually involves iterative design and testing cycles [30,99] with associated formative and summative usability evaluation [45]. Across the life of mHealth development, iterative cycles are likely to span different stages of development and be undertaken in different clinical contexts [54,102]. Integrating usability assessment into this process requires instruments that are generic enough to capture user responses to a wide variety of mHealth strategies but also fine-grained enough to possess sufficient responsiveness [96].

Finally, with regard to the argument of lacking psychometric assessment, none of the preexisting mHealth usability assessment instruments referenced as part of the literature included in this umbrella review appear to have been informed by a breadth of cultural perspective or undergone cross-cultural validity testing. Given the global potential of mHealth to address inequities in access to and outcomes from rehabilitation [5,6], it is particularly important to establish cross-cultural validity of the usability assessment instruments employed in mHealth development. In addition, with the pervasiveness of technology, there is a certain element of unpredictability of the context in which mHealth tools will be trialed and used “in the wild” [103,104]. For that reason, an alternative argument could be made for innovative, culturally responsive methodology for mHealth tool design including usability testing [105]. A key difference in such attempts is user participation at multiple stages of development and responsiveness to expanding the stages of development as guided by stakeholders. This process likely includes constant negotiation and may be resource heavy but is arguably needed if the aim is to create mHealth solutions impacting indigenous outcomes, for example [74,106].

Considering the identified issues, including lack of theoretical grounding, common use of custom-made assessment instruments, and the scarcity of psychometric data for widely used mHealth usability assessment instruments, multimethod usability assessment appears paramount. This is consistent with recommendations made by a number of research groups [13,44,102,107] and reinforces the argument often advanced in favor of Ecological Momentary Assessment approaches, which are recognized for their advantage over retrospective assessment [97]. It is therefore proposed that standards be developed that specify the time points in the mHealth life cycle at which usability assessment is completed, with an emphasis on what methods to use. Moreover, these standards should mandate that individual assessment instruments are grounded in a theoretical framework and possess a minimum threshold for psychometric properties [53,108].

### Recommendations

The establishment of a universal usability scoring system or algorithm would further facilitate the integration of these assessments into an overall framework [109]. It has been observed that, at present, less than half of existing evaluation frameworks include such a scoring system, but that such systems could support funding decisions [29] and advance the vision of prescribable mHealth apps [10]. Although technological advancement often outpaces academic enquiry necessitating new approaches to mHealth evaluation frameworks [110], usability factors are enduring [16] and investing resources into establishing standards will therefore be valuable.

### Limitations

In the context of an area of practice where the lines between commercial and academic work are blurred and usability assessment constitutes a common practice in the global commercial environment [111], this umbrella review is limited to only including English language systematic reviews published within the academic literature indexed in the databases included. Furthermore, the quality of the included systematic reviews was found to be limited, and the fit of the AMSTAR 2 tool with methodological papers is not perfect. However, AMSTAR 2 could be argued to be more detailed than instruments developed for umbrella reviews specifically [31], and, in line with the AMSTAR 2 recommendations [39], the authors modified the list of critical criteria to reflect the specific aim of the overview. Finally, with regard to the review’s methodology, 2 limitations are of note. First, although the search syntax for this umbrella review included the keyword “physical exercise,” for pragmatic reasons, no validation step was included to confirm that all mHealth tools examined as part of the primary studies included within the systematic reviews included a physical exercise component. Regardless, the observations presented here are valid for mHealth tools for rehabilitation overall and provide valuable guidance to developers, researchers, and clinicians. Second, for practical reasons, data selection could only be performed by the primary author (SH) with a subset of articles being screened by a second author (VS). However, agreement on study selection was high (>80%), supporting the quality of the review.

### Conclusions

There is considerable variety in approaches to and instruments for the assessment of usability in mHealth for rehabilitation, many of which lack theoretical foundation. Clinicians are therefore advised to critically evaluate mHealth literature and solutions, paying particular attention to the population in which usability testing was performed and the specific usability assessment instruments were employed. Future research efforts should be focused on producing high-quality systematic reviews and psychometric evaluations of usability assessment instruments. A collaborative effort between researchers, designers, and developers is essential to establish mHealth tool development standards. These standards should emphasize the incorporation of usability assessment instruments underpinned by a robust theoretical base. This umbrella review represents a valuable reference tool in this endeavor. Inclusion of multimethod usability assessment within the wider mHealth development cycle could also be part of these standards, which will ensure that we can capitalize on the widely heralded promise of mHealth to promote access to and outcomes from rehabilitation.

## Data Availability

Lead authors of protocols of systematic reviews were also contacted. If preprint manuscripts were available, they were included in this umbrella review. Systematic reviews not able to be obtained through the authors of the protocol were listed separately for the purpose of the overview [33]. All data accessed and created as part of this umbrella review are included as part of this paper in Tables S2-S5 in Multimedia Appendix 1.

## Appendix

Multimedia Appendix 1 Supplementary files.

## Abbreviations

AMSTAR 2: A Measurement Tool to Assess Systematic Reviews 2
mHealth: mobile health
PACMAD: People At the Centre of Mobile Application Development
PRISMA: Preferred Reporting Items for Systematic Reviews and Meta-Analyses
StArt: State of the Art through Systematic Review
SUS: System Usability Scale
USE: Usefulness, Satisfaction, and Ease of Use

## References

[1] Istepanian RSH, Alanzi T, Feng DD (2022). Mobile health (m-health): evidence-based progress or scientific retrogression. Biomedical Information Technology 2nd ed.

[2] Istepanian RSH, Laxminarayan S, Pattichis CS, Mecheli-Tzanakou E (2006). M-health: emerging mobile health systems. Topics in Biomedical Engineering.

[3] Cao J, Lim Y, Sengoku S, Guo X, Kodama K (2021). Exploring the shift in international trends in mobile health research from 2000 to 2020: bibliometric analysis. JMIR Mhealth Uhealth.

[4] (2017). mHealth app economics 2017—current status and future trends in mobile health. Research 2 Guidance.

[5] Price M, Yuen EK, Goetter EM, Herbert JD, Forman EM, Acierno R, Ruggiero KJ (2014). mHealth: a mechanism to deliver more accessible, more effective mental health care. Clin Psychol Psychother.

[6] Beratarrechea A, Lee AG, Willner JM, Jahangir E, Ciapponi A, Rubinstein A (2014). The impact of mobile health interventions on chronic disease outcomes in developing countries: a systematic review. Telemed J E Health.

[7] Chu JT, Wang MP, Shen C, Viswanath K, Lam TH, Chan SSC (2017). How, when and why people seek health information online: qualitative study in Hong Kong. Interact J Med Res.

[8] Grundy QH, Wang Z, Bero LA (2016). Challenges in assessing mobile health app quality: a systematic review of prevalent and innovative methods. Am J Prev Med.

[9] Nussbaum R, Kelly C, Quinby E, Mac A, Parmanto B, Dicianno BE (2019). Systematic review of mobile health applications in rehabilitation. Arch Phys Med Rehabil.

[10] Byambasuren O, Sanders S, Beller E, Glasziou P (2018). Prescribable mHealth apps identified from an overview of systematic reviews. NPJ Digit Med.

[11] Maramba I, Chatterjee A, Newman C (2019). Methods of usability testing in the development of eHealth applications: a scoping review. Int J Med Inform.

[12] Fiedler J, Eckert T, Wunsch K, Woll A (2020). Key facets to build up eHealth and mHealth interventions to enhance physical activity, sedentary behavior and nutrition in healthy subjects - an umbrella review. BMC Public Health.

[13] Zapata BC, Fernández-Alemán JL, Idri A, Toval A (2015). Empirical studies on usability of mHealth apps: a systematic literature review. J Med Syst.

[14] Harrison R, Flood D, Duce D (2013). Usability of mobile applications: literature review and rationale for a new usability model. J Interact Sci.

[15] Weichbroth P (2020). Usability of mobile applications: a systematic literature study. IEEE Access.

[16] Albert B, Tullis T (2013). Introduction. Measuring the User Experience: Collecting, Analyzing, and Presenting Usability Metrics.

[17] Nielsen J (2019). Defining usability. User Experience Re-mastered: Your Guide to Getting the Right Design.

[18] (2022). Ergonomics of human-system interaction—part 11: usability: definitions and concepts. ISO.

[19] Bevan N, Carter J, Harker S (2015). ISO 9241-11 revised: what have we learnt about usability since 1998?. Lecture Notes in Computer Science (Lecture Notes in Artificial Intelligence and Lecture Notes in Bioinformatics 9169).

[20] (2022). ISO/IEC 25010. ISO 25000 software and data quality. ISO.

[21] Nielsen J (1994). Usability Engineering.

[22] Alturki R, Gay V (2017). Usability testing of fitness mobile application: methodology and quantitative results.

[23] Wildenbos GA, Peute L, Jaspers M (2018). Aging barriers influencing mobile health usability for older adults: a literature based framework (MOLD-US). Int J Med Inform.

[24] National Health Service (2018). Digital assessment questionnaire V2.1. NHS Digital.

[25] (2021). Guidance on applying human factors and usability engineering of medical devices including drug-device combination products in Great Britain. MHRA.

[26] (2022). ORCHA.

[27] (2022). Health navigator app library. New Zealand Ministry of Health.

[28] (2017). Health applications assessment guidance. New Zealand Ministry of Health.

[29] Moshi MR, Tooher R, Merlin T (2018). Suitability of current evaluation frameworks for use in the health technology assessment of mobile medical applications: a systematic review. Int J Technol Assess Health Care.

[30] Bonten TN, Rauwerdink A, Wyatt JC, Kasteleyn MJ, Witkamp L, Riper H, van Gemert-Pijnen LJ, Cresswell K, Sheikh A, Schijven MP, Chavannes NH (2020). Online guide for electronic health evaluation approaches: systematic scoping review and concept mapping study. J Med Internet Res.

[31] Aromataris E, Fernandez R, Godfrey CM, Holly C, Khalil H, Tungpunkom P (2015). Summarizing systematic reviews: methodological development, conduct and reporting of an umbrella review approach. Int J Evid Based Healthc.

[32] Pollock M, Fernandes RM, Pieper D, Tricco AC, Gates M, Gates A, Hartling L (2019). Preferred Reporting Items for Overviews of Reviews (PRIOR): a protocol for development of a reporting guideline for overviews of reviews of healthcare interventions. Syst Rev.

[33] Pollock M, Fernandes RM, Becker LA, Pieper D, Hartling L (2021). Chapter V: overviews of reviews. Cochrane Handbook for Systematic Reviews of Interventions.

[34] Hunt H, Pollock A, Campbell P, Estcourt L, Brunton G (2018). An introduction to overviews of reviews: planning a relevant research question and objective for an overview. Syst Rev.

[35] Silva C, Zamboni A, Hernandes E, Di TA, Belgamo A, Fabbri S (2022). State of the art through systematic review. LaPES.

[36] Davis TL, DiClemente R, Prietula M (2016). Taking mHealth forward: examining the core characteristics. JMIR Mhealth Uhealth.

[37] Chandler J, Cumpston M, Thomas J, Higgins J, Deeks J, Clarke M, Higgins JPT, Green S, Chandler J, Cumpston M, Page M, Welch V (2022). Chapter I: introduction. Cochrane Handbook for Systematic Reviews of Interventions Version 6.

[38] Liberati A, Altman DG, Tetzlaff J, Mulrow C, Gøtzsche PC, Ioannidis JPA, Clarke M, Devereaux PJ, Kleijnen J, Moher D (2009). The PRISMA statement for reporting systematic reviews and meta-analyses of studies that evaluate healthcare interventions: explanation and elaboration. BMJ.

[39] Shea BJ, Reeves BC, Wells G, Thuku M, Hamel C, Moran J, Moher D, Tugwell P, Welch V, Kristjansson E, Henry DA (2017). AMSTAR 2: a critical appraisal tool for systematic reviews that include randomised or non-randomised studies of healthcare interventions, or both. BMJ.

[40] Gates M, Gates A, Guitard S, Pollock M, Hartling L (2020). Guidance for overviews of reviews continues to accumulate, but important challenges remain: a scoping review. Syst Rev.

[41] Bougioukas KI, Vounzoulaki E, Mantsiou CD, Savvides ED, Karakosta C, Diakonidis T, Tsapas A, Haidich A (2021). Methods for depicting overlap in overviews of systematic reviews: an introduction to static tabular and graphical displays. J Clin Epidemiol.

[42] Hennessy EA, Johnson BT (2020). Examining overlap of included studies in meta-reviews: guidance for using the corrected covered area index. Res Synth Methods.

[43] Lewis JR (2018). The system usability scale: past, present, and future. Int J Hum Comput Interact Taylor & Francis.

[44] Georgsson M (2020). A review of usability methods used in the evaluation of mobile health applications for diabetes. Stud Health Technol Inform.

[45] Inal Y, Wake JD, Guribye F, Nordgreen T (2020). Usability evaluations of mobile mental health technologies: systematic review. J Med Internet Res.

[46] Ng MM, Firth J, Minen M, Torous J (2019). User engagement in mental health apps: a review of measurement, reporting, and validity. Psychiatr Serv.

[47] Azad-Khaneghah P, Neubauer N, Miguel Cruz A, Liu L (2021). Mobile health app usability and quality rating scales: a systematic review. Disabil Rehabil Assist Technol.

[48] Vera F, Noël R, Taramasco C (2019). Standards, processes and instruments for assessing usability of health mobile apps: a systematic literature review. Stud Health Technol Inform.

[49] Saeed N, Manzoor M, Khosravi P (2020). An exploration of usability issues in telecare monitoring systems and possible solutions: a systematic literature review. Disabil Rehabil Assist Technol.

[50] Nouri R, Kalhori SRN, Ghazisaeedi M, Marchand G, Yasini M (2018). Criteria for assessing the quality of mHealth apps: a systematic review. J Am Med Inform Assoc.

[51] Muro-Culebras A, Escriche-Escuder A, Martin-Martin J, Roldán-Jiménez C, De-Torres I, Ruiz-Muñoz M, Gonzalez-Sanchez M, Mayoral-Cleries F, Biró A, Tang W, Nikolova B, Salvatore A, Cuesta-Vargas AI (2021). Tools for evaluating the content, efficacy, and usability of mobile health apps according to the consensus-based standards for the selection of health measurement instruments: systematic review. JMIR Mhealth Uhealth.

[52] Wakefield BJ, Turvey CL, Nazi KM, Holman JE, Hogan TP, Shimada SL, Kennedy DR (2017). Psychometric properties of patient-facing ehealth evaluation measures: systematic review and analysis. J Med Internet Res.

[53] Kien C, Schultes MT, Szelag M, Schoberberger R, Gartlehner G (2018). German language questionnaires for assessing implementation constructs and outcomes of psychosocial and health-related interventions: a systematic review. Implement Sci.

[54] Niknejad N, Ismail W, Bahari M, Nazari B (2021). Understanding telerehabilitation technology to evaluate stakeholders' adoption of telerehabilitation services: a systematic literature review and directions for further research. Arch Phys Med Rehabil.

[55] Canadian Institutes of Health Research (CIHR) (2016). Mental health apps: how to make an informed choice. Mental Health Commission of Canada (MHCC).

[56] Brooke J (1996). SUS: a 'Quick and Dirty' usability scale. Usability Evaluation In Industry.

[57] Lewis JR (1995). IBM computer usability satisfaction questionnaires: psychometric evaluation and instructions for use. Int J Hum-Comput Interact.

[58] Lund A (2001). Measuring usability with the USE questionnaire. Usability Interface.

[59] van Singer M, Chatton A, Khazaal Y (2015). Quality of smartphone apps related to panic disorder. Front Psychiatry.

[60] Lewis JR (1991). An after-scenario questionnaire for usability studies: psychometric evaluation over three trials. ACM SIGCHI Bulletin.

[61] McNiel P, McArthur EC (2016). Evaluating health mobile apps: information literacy in undergraduate and graduate nursing courses. J Nurs Educ.

[62] Huis in 't Veld RMHA, Kosterink SM, Barbe T, Lindegård A, Marecek T, Vollenbroek-Hutten MMR (2010). Relation between patient satisfaction, compliance and the clinical benefit of a teletreatment application for chronic pain. J Telemed Telecare.

[63] Baumel A, Faber K, Mathur N, Kane JM, Muench F (2017). Enlight: a comprehensive quality and therapeutic potential evaluation tool for mobile and web-based ehealth interventions. J Med Internet Res.

[64] Yen P, Wantland D, Bakken S (2010). Development of a customizable health IT usability evaluation scale. AMIA Annu Symp Proc.

[65] Brown W, Yen P, Rojas M, Schnall R (2013). Assessment of the health IT usability evaluation model (Health-ITUEM) for evaluating mobile health (mHealth) technology. J Biomed Inform.

[66] Taki S, Campbell KJ, Russell CG, Elliott R, Laws R, Denney-Wilson E (2015). Infant feeding websites and apps: a systematic assessment of quality and content. Interact J Med Res.

[67] Gediga G, Hamborg KC, Düntsch I (1999). The IsoMetrics usability inventory: An operationalization of ISO 9241-10 supporting summative and formative evaluation of software systems. Behaviour & Information Technology.

[68] Grau I, Kostov B, Gallego JA, Grajales Iii F, Fernández-Luque L, Sisó-Almirall A (2016). Assessment method for mobile health applications in Spanish: the iSYScore index. Semergen.

[69] Jin M, Kim J (2015). Development and evaluation of an evaluation tool for healthcare smartphone applications. Telemed J E Health.

[70] Davis FD (1989). Perceived usefulness, perceived ease of use, and user acceptance of information technology. MIS Quarterly.

[71] Stoyanov SR, Hides L, Kavanagh DJ, Zelenko O, Tjondronegoro D, Mani M (2015). Mobile app rating scale: a new tool for assessing the quality of health mobile apps. JMIR Mhealth Uhealth.

[72] Stoyanov SR, Hides L, Kavanagh DJ, Wilson H (2016). Development and validation of the user version of the mobile application rating scale (uMARS). JMIR Mhealth Uhealth.

[73] Hart SG (2006). Nasa-task load index (NASA-TLX); 20 years later. Proc Human Fact Ergon Soc Annu Meet.

[74] McMillan B, Hickey E, Patel MG, Mitchell C (2016). Quality assessment of a sample of mobile app-based health behavior change interventions using a tool based on the National Institute of Health and Care Excellence behavior change guidance. Patient Educ Couns.

[75] Price M, Sawyer T, Harris M, Skalka C (2016). Usability evaluation of a mobile monitoring system to assess symptoms after a traumatic injury: a mixed-methods study. JMIR Ment Health.

[76] Lewis JR (2016). Psychometric evaluation of the post-study system usability questionnaire: the PSSUQ. Proc Hum Fact Soc Annu Meet.

[77] Loy JS, Ali EE, Yap KY (2016). Quality assessment of medical apps that target medication-related problems. J Manag Care Spec Pharm.

[78] Martínez-Pérez B, de la Torre-Díez I, Candelas-Plasencia S, López-Coronado M (2013). Development and evaluation of tools for measuring the quality of experience (QoE) in mHealth applications. J Med Syst.

[79] Chin JP, Diehl VA, Norman KL (1988). Development of an instrument measuring user satisfaction of the human-computer interface.

[80] Silberg WM, Lundberg GD, Musacchio RA (1997). Assessing, controlling, and assuring the quality of medical information on the Internet: caveant lector et viewor—let the reader and viewer beware. JAMA.

[81] Kirakowski J, Corbett M (1993). SUMI: the software usability measurement inventory. Br J Educ Technol.

[82] Parmanto B, Pulantara IW, Schutte JL, Saptono A, McCue MP (2013). An integrated telehealth system for remote administration of an adult autism assessment. Telemed J E Health.

[83] Bakken S, Grullon-Figueroa L, Izquierdo R, Lee N, Morin P, Palmas W, Teresi J, Weinstock RS, Shea S, Starren J (2006). Development, validation, and use of English and Spanish versions of the telemedicine satisfaction and usefulness questionnaire. J Am Med Inform Assoc.

[84] Zhou L, Bao J, Setiawan IMA, Saptono A, Parmanto B (2019). The mHealth app usability questionnaire (MAUQ): development and validation study. JMIR Mhealth Uhealth.

[85] Suso-Martí L, La Touche R, Herranz-Gómez A, Angulo-Díaz-Parreño S, Paris-Alemany A, Cuenca-Martínez F (2021). Effectiveness of telerehabilitation in physical therapist practice: an umbrella and mapping review with meta-meta-analysis. Phys Ther.

[86] Marcolino MS, Oliveira JAQ, D'Agostino M, Ribeiro AL, Alkmim MBM, Novillo-Ortiz D (2018). The impact of mHealth interventions: systematic review of systematic reviews. JMIR Mhealth Uhealth.

[87] Mönninghoff A, Kramer JN, Hess AJ, Ismailova K, Teepe GW, Tudor Car L, Müller-Riemenschneider F, Kowatsch T (2021). Long-term effectiveness of mHealth physical activity interventions: systematic review and meta-analysis of randomized controlled trials. J Med Internet Res.

[88] Elavsky S, Knapova L, Klocek A, Smahel D (2019). Mobile health interventions for physical activity, sedentary behavior, and sleep in adults aged 50 years and older: a systematic literature review. J Aging Phys Act.

[89] Direito A, Carraça E, Rawstorn J, Whittaker R, Maddison R (2017). mHealth technologies to influence physical activity and sedentary behaviors: behavior change techniques, systematic review and meta-analysis of randomized controlled trials. Ann Behav Med.

[90] Koumpouros Y, Georgoulas A (2020). Inform Health Soc Care.

[91] Rabinowitz AR, Juengst SB (2022). Introduction to topical Issue on mHealth for brain injury rehabilitation. J Head Trauma Rehabil.

[92] Baumgartner J, Ruettgers N, Hasler A, Sonderegger A, Sauer J (2021). Questionnaire experience and the hybrid system usability scale: using a novel concept to evaluate a new instrument. Int J Hum Comput Stud.

[93] Ovčjak B, Heričko M, Polančič G (2015). Factors impacting the acceptance of mobile data services—a systematic literature review. Comput Human Behav.

[94] Gao M, Kortum P, Oswald FL (2018). Psychometric evaluation of the USE (Usefulness, Satisfaction, and Ease of use) questionnaire for reliability and validity.

[95] Peres SC, Pham T, Phillips R (2013). Validation of the System Usability Scale (SUS): SUS in the Wild. Proc Hum Factors Ergon Soc Annu Meet.

[96] McDowell I (2006). The theoretical and technical foundations of health measurement. Measuring Health.

[97] Demers M, Winstein CJ (2021). A perspective on the use of ecological momentary assessment and intervention to promote stroke recovery and rehabilitation. Top Stroke Rehabil.

[98] Boyd K, Bond R, Vertesi A, Dogan H, Magee J (2019). How people judge the usability of a desktop graphic user interface at different time points: is there evidence for memory decay, recall bias or temporal bias?. Interact Comput.

[99] Alwashmi MF, Hawboldt J, Davis E, Fetters MD (2019). The iterative convergent design for mobile health usability testing: mixed methods approach. JMIR Mhealth Uhealth.

[100] Switzer GE, Wisniewski SR, Belle SH, Dew MA, Schultz R (1999). Selecting, developing, and evaluating research instruments. Soc Psychiatry Psychiatr Epidemiol.

[101] Hughes DJ, Irwing P, Booth T, Hughes DJ (2018). Psychometric validity: establishing the accuracy and appropriateness of psychometric measures. The Wiley Handbook of Psychometric Testing: A Multidisciplinary Reference on Survey, Scale and Test Development.

[102] Zein S, Salleh N, Grundy J (2016). A systematic mapping study of mobile application testing techniques. J Syst Softw.

[103] Brown B, Reeves S, Sherwood S (2011). Into the wild: challenges and opportunities for field trial methods.

[104] Kjeldskov J, Stage J (2004). New techniques for usability evaluation of mobile systems. Int J Hum-Comput Stud.

[105] Rolleston AK, Bowen J, Hinze A, Korohina E, Matamua R (2021). Collaboration in research: weaving Kaupapa Māori and computer science. AlterNative.

[106] Stowell E, Lyson M, Saksono H, Wurth R, Jimison H, Pavel M, Parker A (2018). Designing and evaluating mHealth interventions for vulnerable populations: a systematic review.

[107] Bernhaupt R, Mihalic K, Obrist M (2011). Usability evaluation methods for mobile applications. Handbook of Research on User Interface Design and Evaluation for Mobile Technology.

[108] Lewis CC, Mettert KD, Dorsey CN, Martinez RG, Weiner BJ, Nolen E, Stanick C, Halko H, Powell BJ (2018). An updated protocol for a systematic review of implementation-related measures. Syst Rev.

[109] Oyebode O, Alqahtani F, Orji R (2020). Using machine learning and thematic analysis methods to evaluate mental health apps based on user reviews. IEEE Access.

[110] Quintana Y, Torous J (2020). A framework for evaluation of mobile apps for youth mental health. Homewood Research Institute.

[111] Hosseiniravandi M, Kahlaee AH, Karim H, Ghamkhar L, Safdari R (2020). Home-based telerehabilitation software systems for remote supervising: a systematic review. Int J Technol Assess Health Care.

