# Supplementary material for: Usability Assessment Methods for Mobile Apps for Physical Rehabilitation: Umbrella Review
Source: JMIR Mhealth Uhealth. 2024 Oct 4;12:e49449. doi: 10.2196/49449 (PMC11489792; doi:10.2196/49449)
Supplement: Multimedia Appendix 1 [file mhealth_v12i1e49449_app1.docx]

## Multimedia Appendix 1

Table S1: Search syntax for databases included as part of the present overview

| **PubMed** |
| --- |
| Search: mobile application* OR mobile app AND usab* OR usab* criteria OR usab* evaluat* AND systematic review AND mhealth OR mobile health AND physical activity Filters: Systematic Review, Humans, English, from 2015 - 2022  (((((((("mobile"[All Fields] OR "mobiles"[All Fields]) AND "application*"[All Fields]) OR ("mobile applications"[MeSH Terms] OR ("mobile"[All Fields] AND "applications"[All Fields]) OR "mobile applications"[All Fields] OR ("mobile"[All Fields] AND "app"[All Fields]) OR "mobile app"[All Fields])) AND "usab*"[All Fields]) OR ("usab*"[All Fields] AND ("criteria s"[All Fields] OR "criterias"[All Fields] OR "standards"[MeSH Subheading] OR "standards"[All Fields] OR "criteria"[All Fields])) OR ("usab*"[All Fields] AND "evaluat*"[All Fields])) AND ("systematic review"[Publication Type] OR "systematic reviews as topic"[MeSH Terms] OR "systematic review"[All Fields]) AND ("mhealth s"[All Fields] OR "telemedicine"[MeSH Terms] OR "telemedicine"[All Fields] OR "mhealth"[All Fields])) OR ("telemedicine"[MeSH Terms] OR "telemedicine"[All Fields] OR ("mobile"[All Fields] AND "health"[All Fields]) OR "mobile health"[All Fields])) AND ("exercise"[MeSH Terms] OR "exercise"[All Fields] OR ("physical"[All Fields] AND "activity"[All Fields]) OR "physical activity"[All Fields])) AND ((systematicreview[Filter]) AND (humans[Filter]) AND (2017:2022[pdat]) AND (english[Filter]))  Translations  mobile: "mobile"[All Fields] OR "mobiles"[All Fields]  mobile app: "mobile applications"[MeSH Terms] OR ("mobile"[All Fields] AND "applications"[All Fields]) OR "mobile applications"[All Fields] OR ("mobile"[All Fields] AND "app"[All Fields]) OR "mobile app"[All Fields]  criteria: "criteria's"[All Fields] OR "criterias"[All Fields] OR "standards"[Subheading] OR "standards"[All Fields] OR "criteria"[All Fields]  systematic review: "systematic review"[Publication Type] .or. "systematic reviews as topic"[MeSH Terms] .or. "systematic review"[All Fields]  mhealth: "mhealth's"[All Fields] OR "telemedicine"[MeSH Terms] OR "telemedicine"[All Fields] OR "mhealth"[All Fields]  mobile health: "telemedicine"[MeSH Terms] OR "telemedicine"[All Fields] OR ("mobile"[All Fields] AND "health"[All Fields]) OR "mobile health"[All Fields]  physical activity: "exercise"[MeSH Terms] OR "exercise"[All Fields] OR ("physical"[All Fields] AND "activity"[All Fields]) OR "physical activity"[All Fields] |
| **Cochrane** |
| (((((mobile application* or mobile app) and usab*) or usab* criteria or usab* evaluat*) and systematic review and mhealth) or mobile health).mp. [mp=ti, ab, tx, kw, ct, hw] |
| **IEEE Xplore** |
| "All Metadata":mobile application* OR "All Metadata":mobile app* AND "All Metadata":usab* OR "All Metadata":usab* criteria OR "All Metadata":usab* evaluat* AND "All Metadata":systematic review AND "All Metadata":mhealth OR "All Metadata":mobile health AND "All Metadata":physical exercise” Additional settings: Date restriction 2015-2022; Journals; Publication Topics: healthcare |
| **Epistemonikos** |
| (title:(mobile application* OR mobile app AND usab* OR usab* criteria OR usab* evaluat* AND systematic review AND mhealth OR mobile health AND physical exercise) OR abstract:(mobile application* OR mobile app AND usab* OR usab* criteria OR usab* evaluat* AND systematic review AND mhealth OR mobile health AND physical exercise))  Additional settings: Date restriction 2015-2022; Publication type: Systematic Review |
| **Web of Science** |
| ALL=(mobile application* OR mobile app AND usab* OR usab* criteria OR usab* evaluat* AND systematic review AND mhealth OR mobile health AND physical exercise) and Health Care Sciences Services or Rehabilitation (Research Areas) and Review Articles (Document Types) and English (Languages) |
| **CINAHL Complete** |
| mobile application* OR mobile app* AND usab* OR usab* criteria OR usab* evaluat* AND systematic review AND mhealth OR mobile health AND physical exercise  Additional settings: Limiters - Published Date: 20150101-; English Language; Peer Reviewed; Human; Language: English; Publication Type: Systematic Review; Expanders - Apply related words; Apply equivalent subjects; Search modes - Boolean/Phrase |

Table S2: Citation details of protocol studies for which lead authors were contacted for full-text articles and citation details of publications excluded through full-text screening

| **Authors** | **Title** | **Year** | **Journal** | **Volume** | **Pages** | **DOI** | **ISSN/ISBN** | **Status/Selection** | **Status/Extraction** | **comment** |
| --- | --- | --- | --- | --- | --- | --- | --- | --- | --- | --- |
| Zhou, LM; Bao, J; Parmanto, B | Systematic Review Protocol to Assess the Effectiveness of Usability Questionnaires in mHealth App Studies | 2017 | JMIR Research Protocols | 6 | e151 | 10.2196/resprot.7826 | 1929-0748 | ACCEPTED | CONTACT LEAD AUTHOR | unable to retrieve full text article |
| Radbron, E; Wilson, V; McCance, T; Middleton, R | The Use of Data Collected From mHealth Apps to Inform Evidence-Based Quality Improvement: An Integrative Review | 2019 | Worldviews on Evidence-Based Nursing | 16 | 70 - 77 | 10.1111/wvn.12343 | 1545-102X | ACCEPTED | REJECTED | excluded due to no mention of usability assessment methods |
| Binyamin, SS; Zafar, B A | Proposing a mobile apps acceptance model for users in the health area: A systematic literature review and meta-analysis | 2021 | Health Informatics Journal | 27 | 1 - 27 | 10.1177/1460458220976737 | 1460-4582 | ACCEPTED | REJECTED | excluded due to no mention of usability assessment methods |
| Baumel, A; Birnbaum, M; Sucala, M | A Systematic Review and Taxonomy of Published Quality Criteria Related to the Evaluation of User-Facing eHealth Programs | 2017 | Journal of Medical Systems | 41 | 1 - 7 | 10.1007/s10916-017-0776-6 | 0148-5598 | ACCEPTED | REJECTED | confirms that usability constitutes a common factor tracked as part of quality assessment of many eHealth programs (see Table 2) no information about usability assessment scales or checklists beyond the binary (presence yes/no) (Table 4- Supplementary Files) |

| **Authors** | **Title** | **Year** | **Journal** | **Volume** | **Pages** | **DOI** | **ISSN/ISBN** | **Status/Selection** | **Status/Extraction** | **comment** |
| --- | --- | --- | --- | --- | --- | --- | --- | --- | --- | --- |
| Lee, S; Lee, Y; Lee, S; Islam, S; Mohammed S; Kim, S-Y | Toward Developing a Standardized Core Set of Outcome Measures in Mobile Health Interventions for Tuberculosis Management: Systematic Review | 2019 | JMIR mHealth and uHealth | 7 | no page numbers | 10.2196/12385 |  | ACCEPTED | REJECTED | provides broad overview of (definitions and types of) outcome measures examined regarding mHealth for tuberculosis without a review of usability assessment measures |
| Zischke, C; Simas, V; Hing, W; Milne, N; Spittle, A; Pope, R | The utility of physiotherapy assessments delivered by telehealth: A systematic review | 2021 | Journal of global health | 11 | no page numbers | 10.7189/jogh.11.04072 |  | ACCEPTED | REJECTED | provides a high-level overview of the portion of the literature evaluating mHealth systems (e.g., cameras, videoconference) for physiotherapy assessment including usability testing but no review of specific usability assessment methods |
| Aji, M; Gordon, C; Stratton, E; Calvo, RA; Bartlett, D; Grunstein, R; Glozier, N. | Framework for the Design Engineering and Clinical Implementation and Evaluation of mHealth Apps for Sleep Disturbance: Systematic Review | 2021 | Journal of Medical Internet Research | 23 | no page numbers | 10.2196/24607 | 1438-8871 | ACCEPTED | REJECTED | provides a high-level overview of the portion of the literature evaluating mHealth solutions for sleep disturbance including usability testing but no review of specific usability assessment methods |
| Larbi, D; Randine, P; Arsand, E; Antypas, K; Bradway, M; Gabarron, E | Methods and Evaluation Criteria for Apps and Digital Interventions for Diabetes Self-Management: Systematic Review | 2020 | Journal of Medical Internet Research | 22 | no page numbers | 10.2196/18480 | 1438-8871 | ACCEPTED | REJECTED | provides a high-level overview of the portion of the literature evaluating mHealth solutions for diabetes including usability testing but no review of specific usability assessment methods |
| **Authors** | **Title** | **Year** | **Journal** | **Volume** | **Pages** | **DOI** | **ISSN/ISBN** | **Status/Selection** | **Status/Extraction** | **comment** |
| Moshi, MR; Tooher, R; Merlin, T | Suitability of current evaluation frameworks for use in the heath technology assessment of mobile medical applications: A systematic review | 2018 | International Journal of Technology Assessment in Health Care | 34 | 464-475 | 10.1017/S026646231800051X | 1471-6348 | ACCEPTED | REJECTED | no review of specific usability assessment methods; provides summary of existing health technology evaluation frameworks and charts simple presence/absence of effectiveness as factor included; main focus on safety/potential harm |
| Bonten, TN ; Rauwerdink, A; Wyatt, JC; Kasteleyn, MJ; Witkamp, L; Riper, H; van Gemert-Pijnen, LJ; Cresswell, K; Sheikh, A; Schijven, MP; Chavannes, NH; EHlth Evaluation Res Grp | Online Guide for Electronic Health Evaluation Approaches: Systematic Scoping Review and Concept Mapping Study | 2020 | Journal of Medical Internet Research | 22 | no page numbers | 10.2196/17774 | 1438-8871 | ACCEPTED | REJECTED | no review of specific usability assessment methods included; provides a high level overview of the portion of the mHealth literature that includes usability testing (as part of effectiveness/efficacy) and performed a concept mapping exercise with developers to create an "eHealth methodology guide" open source document for researchers |

| **Authors** | **Title** | **Year** | **Journal** | **Volume** | **Pages** | **DOI** | **ISSN/ISBN** | **Status/Selection** | **Status/Extraction** | **comment** |
| --- | --- | --- | --- | --- | --- | --- | --- | --- | --- | --- |
| Yoshida, Y; Patil, SJ; Brownson, RC; Boren, SA; Kim, M; Dobson, R; Greenwood, DA; Torbjornsen, A; Ramachandran, A; Masi, C; Fonseca, VA; Simoes, EJ | Using the RE-AIM framework to evaluate internal and external validity of mobile phone-based interventions in diabetes self-management education and support | 2020 | Journal of the American Medical Informatics Association | 27 | 946-956 | 10.1093/jamia/ocaa041 | 1527-974X | ACCEPTED | REJECTED | provides a high-level overview of the portion of the mHealth literature that includes usability testing (as part of effectiveness/efficacy) but does not include a review of specific usability assessment methods |
| Hosseiniravand, M; Kahlaee, A; Karim, H; Ghamkhar, L; Safdari, R | Home-based telerehabilitation software systems for remote supervising: a systematic review | 2020 | International Journal of Technology Assessment in Health Care | 36 | 113-125 | 10.1017/S0266462320000021 | 1471-6348 | ACCEPTED | REJECTED | provides a high-level overview of the portion of the mHealth literature that includes usability testing but does not include a review of specific usability assessment methods |

| **Authors** | **Title** | **Year** | **Journal** | **Volume** | **Pages** | **DOI** | **ISSN/ISBN** | **Status/Selection** | **Status/Extraction** | **comment** |
| --- | --- | --- | --- | --- | --- | --- | --- | --- | --- | --- |
| Kumar, S; Southard, P B; White, M | Telemedicine: determining "critical to quality" characteristics for a healthcare service system design based on a survey of physical rehabilitation providers | 2016 | IEEE Engineering Management Review | 44 | 41-55 | 10.1109/EMR.2016.2568959 | 1937-4178 | ACCEPTED | REJECTED | study reports on results of survey of physical rehabilitation providers rather than survey of literature (i.e., Systematic Review) |
| Grundy, Quinn H.; Wang, Zhicheng; Bero, Lisa A. | Challenges in Assessing Mobile Health App Quality: A Systematic Review of Prevalent and Innovative Methods | 2016 | American Journal of Preventive Medicine | 51 | 1051-1059 | 10.1016/j.amepre.2016.07.009 | 0749-3797 | ACCEPTED | REJECTED | provides a high-level overview of the portion of the mHealth literature that includes usability testing but does not include a review of specific usability assessment methods |
| Boris Ovcˇjak, M; Hericˇko, G; Polancˇic | Factors impacting the acceptance of mobile data services – A systematic literature review | 2015 | Computers in Human Behavior | 53 | 24-47 | 10.1016/j.chb.2015.06.013 | 0747-5632 | ACCEPTED | REJECTED | examined mobile service categories other than health (e.g., entertainment, communication, transaction), the quality of that literature and its reference to acceptance models |
| Muntaner, A; Vidal-Conti, J; Palou, P | Increasing physical activity through mobile device interventions: A systematic review | 2016 | Health Informatics Journal | 22 | 451-469 | 10.1177/1460458214567004 | 1460-4582 | ACCEPTED | REJECTED | excluded due to no mention of usability assessment methods |

Table S3: Descriptive summary of reviews included within the present overview

|  | Date range | Population | Intervention | Outcome | Inclusion criteria | Exclusion criteria | Number of studies screened (additional studies identified by reviewing references) | Number of primary studies included | Usability framework/model referenced (Yes- detail; No) | Notes | AMSTAR2 confidence rating |
| --- | --- | --- | --- | --- | --- | --- | --- | --- | --- | --- | --- |
| Azad-Khaneghah et al. (2021) | 2000- August 2018 | Patients, clinicians, target users | mHealth | Usability rating scales, quality assessment tools | Any language; peer-reviewed, conference or grey literature; reporting scales/questionnaires for usability assessment of health-related applications | No full text; non-health applications; no reporting of usability scales; studies about stand-alone websites | 3677 (3) | 87 | Yes, MHCC, CIHR | Emphasize need for usability assessment to be based off theoretical framework | Low |
| Georgsson (2020) | 2015-2020 | Diabetes patients | mHealth | Usability evaluation/tests | English language; empirical studies; usability evaluation studies; with published outcomes | Study protocols | 131 | 34 | Yes- implied ISO in including ISO definition of ‘usability’ | Emphasize need for >1 usability assessment method; recommendation that one measure constitutes a tool with established psychometric properties | critically low |

|  | Date range | Population | Intervention | Outcome | Inclusion criteria | Exclusion criteria | Number of studies screened (additional studies identified by reviewing references) | Number of primary studies included | Usability framework/model referenced (Yes- detail; No) | Notes | AMSTAR2 confidence rating |
| --- | --- | --- | --- | --- | --- | --- | --- | --- | --- | --- | --- |
| Inal et al. (2020) | 2001-2018 | mHealth users living with mental health concern, mental illness, mental disorder, psychiatric illness | mHealth, eHealth, internet cCBT | Usability evaluation, assessment measure, test, testing, heuristics | Empirical studies with focus on usability evaluation of mobile digital mental health interventions; English language | Non-English language sources; no full text or conference paper/abstract or study protocol; limited to mobile use such as SMS, web browser or sensors | 299 | 42 | Nielsen | Use of adaptation of standardized usability questionnaires widespread highlighting need for standardized mHealth usability measure | critically low |
| Kien et al. (2018) | 1985-August 2017 | Health-related innovation/intervention | Quality assessment tools | Psychometric properties | Peer-reviewed sources reporting on quantitative instrument assessing health-related intervention assessing one or more CFIR/IOF constructs and including assessment of at least one psychometric property | Sources without test-theoretical assessment of non-health related tools applied to non-German speaking population; no full text | 1668 | 38 | Reference to theoretical foundations of each of the included instruments | There is a lack of psychometrically strong intervention evaluation tools particularly regarding internal consistency structural and criterion validity | moderate |
|  | Date range | Population | Intervention | Outcome | Inclusion criteria | Exclusion criteria | Number of studies screened (additional studies identified by reviewing references) | Number of primary studies included | Usability framework/model referenced (Yes- detail; No) | Notes | AMSTAR2 con-fidence rating |
| Muro-Culebras et al. (2021) | none | mHealth applications | Quality assessment tools | Psychometric properties | Studies validating quality assessment tools for mHealth apps | Studies not including psychometric validation | 2831 | 10 | No explicit reference | Report general lack of psychometric validation of usability assessment measures; Suggest MARS as candidate for continued development and psychometric evaluation | Low |
| Ng et al. (2019) | Not given | mHealth users living with mental health concern, mental illness, mental disorder, psychiatric illness | mHealth | User engagement indicators (usability, satisfaction, acceptability, feasibility) | Empirical study of mobile application designed for population of interest with conclusion about usability and minimum duration of 7 days | Non-English publications including reviews, conference reports, protocols, dissertation focusing on other technologies or user groups | 882 | 40 | None given | Report great diversity in definitions and measures of usability and call for creation of reporting standards | critically low |
|  | Date range | Population | Intervention | Outcome | Inclusion criteria | Exclusion criteria | Number of studies screened (additional studies identified by reviewing references) | Number of primary studies included | Usability framework/model referenced (Yes- detail; No) | Notes | AMSTAR2 confidence rating |
| Niknejad et al. (2021) | 1998-August 2020 | mHealth users | TeleRehab systems | Usability, effectiveness (user satisfaction; user acceptance/adherence; readiness; motivation/awareness; user perception, perspective & experience) | English  Empirical studies  Experimental studies; Studies fulfilling custom-made quality assessment criteria | Non-English language studies which did not fulfil custom-made quality assessment | 357 | 133 | Reference to theoretical foundations of the included instruments when given in primary studies | Highlight that evaluation of majority of technologies examined did not include acceptance and usability among end users | critically low |
| Nouri et al. (2018) | 2008-2016 | Patients, clinicians, target users | mHealth | mHealth evaluation methods/ tools | English language studies that provide evaluation tool/method for mHealth | Non-English language sources examining mobile apps not related to medicine/health or focusing only on design and development of mHealth app | 851  (2) | 23 | None | Framework of 7 categories of assessment criteria developed; usability was one category under which 13 evaluation criteria were re-classified | critically low |
|  | Date range | Population | Intervention | Outcome | Inclusion criteria | Exclusion criteria | Number of studies screened (additional studies identified by reviewing references) | Number of primary studies included | Usability framework/model referenced (Yes- detail; No) | Notes | AMSTAR2 con-fidence rating |
| Saeed et al. (2020) | 1999-2017 | Users affected by disability/disease targeted by applications | Applications | Usability features | English language sources; Focus on telecare monitoring systems; focus on usability; discussion of end users; peer-reviewed journal articles or conference proceedings | Non-English language sources; systematic reviews; systematic mapping studies | 453 | 16 | ISO 9421-11; ISO/IEC 9126-1 | Emphasise importance of involvement of end users in design and development of mHealth solutions to enhance usability | critically low |
| Vera et al. (2019) | 2000-2017 | mHealth | Use/usability of health information systems | Not specified | English language; assessment of health-related mobile apps; quality/usability assessment | Non-English sources; studies not relating to mobile apps or apps outside of health | 493 | 10 | ISO 9241-11:2018; ISO 9241-12:1998; ISO/IEC 9126-1 | Identify PSSUQ, SUS and MARS as most referenced measures; citation details of included studies not part of the publication | critically low |

|  | Date range | Population | Intervention | Outcome | Inclusion criteria | Exclusion criteria | Number of studies screened (additional studies identified by reviewing references) | Number of primary studies included | Usability framework/model referenced (Yes- detail; No) | Notes | AMSTAR2 confidence rating |
| --- | --- | --- | --- | --- | --- | --- | --- | --- | --- | --- | --- |
| Wakefield et al. (2017) | -June 2014 | mHealth applications | Quality assessment tools | Psychometric properties | English language sources describing development or use of evaluation measure including description of one or more psychometric properties | Non-English language sources, no full text; no inclusion of psychometric properties; disease-specific assessments; clinician focused tools | 32743 | 23 | Reference to theoretical foundations of each of the included instruments | Emphasize need for psychometrically strong measures evaluating mHealth applications | Low |
| Zapata et al. (2015) | 2000- March 2014 | Patients, clinicians, target users | mHealth | Usability assessment | English language empirical investigation of end-user usability of mHealth tool accessed via smartphone/tablet | Non-English language sources with no full text, focusing on PDA or feature phone and/or features other than mobile applications | 642 | 22 | ISO/IEC 9126-1 | Recommend improvement of usability assessment through automated processes and combination of at least two assessment methods | critically low |

AMIA: American Medical Informatics Association; AMSTAR2 confidence ration: low= 1 critical weakness; critically low= 2 or more critical weaknesses; CIHR: Canadian Institutes of Health Research; CFIR: Consolidated Framework for Implementation Research; HIMMS: Healthcare Information and Management Systems Society; IOF: Implementation Outcomes Framework; MARS: Mobile Application Rating Scale; MHCC: Mental Health Commission of Canada; PSSUQ: Post-Study System Usability Questionnaire: SUS: System Usability Scale

Table S4: Custom usability measures identified by systematic reviews included within the present overview

|  |  | Systematic Review | | | | | | | | | | |
| --- | --- | --- | --- | --- | --- | --- | --- | --- | --- | --- | --- | --- |
| Count | Primary Study | Azad-Khaneghah et al. (2021) | Georgsson (2020) | Inal et al. (2020) | Muro-Culebras et al. (2021) | Ng et al. (2019) | Niknejad et al. (2021) | Nouri et al. (2018) | Saeed et al. (2020) | Vera et al. (2019) | Wakefield et al. (2017) | Zapata et al. (2015) |
| 1 | Akinola et al. (2019) | Yes | No | No | No | No | No | No | No | No | No | No |
| 2 | Al Ayubi et al. (2014) | Yes | No | No | No | No | No | No | No | No | No | No |
| 3 | Albanese-O’Neill et al. (2019) | No | Yes | No | No | No | No | No | No | No | No | No |
| 4 | Aljaber et al. (2016) | No | No | No | No | No | No | No | No | Yes | No | No |
| 5 | Anderson et al. (2016) | No | No | No | No | No | No | Yes | No | No | No | No |
| 6 | Arnhold et al. (2014) | Yes | No | No | No | No | No | No | No | No | No | No |
| 7 | Atkinson (2007) | No | No | No | No | No | No | No | No | No | Yes | No |
| 8 | Auger et al. (2014) | No | No | Yes | No | No | No | No | No | No | No | No |
| 9 | Bauer et al. (2018) | No | No | No | No | Yes | No | No | No | No | No | No |
| 10 | Ben-Zeev et al (2016) | No | No | No | No | Yes | No | No | No | No | No | No |
| 11 | Ben-Zeev et al. (2018) | No | No | No | No | Yes | No | No | No | No | No | No |
| 12 | Boisseau et al. (2017) | No | No | No | No | Yes | No | No | No | No | No | No |
| 13 | Boman eta al. (2015) | No | No | Yes | No | No | No | No | No | No | No | No |
| 14 | Chenchen et al. (2017) | Yes | No | No | No | No | No | No | No | No | No | No |
| 15 | Cho et al. (2014) | Yes | No | No | No | No | No | No | No | No | No | No |
| 16 | Connelly et al. (2016) | No | No | Yes | No | No | No | No | No | No | No | No |
| 17 | Cruz Zapata et al. (2015) | No | No | No | No | No | No | Yes | No | No | No | No |
| 18 | de la Torre Diez et al. (2017) | Yes | No | No | No | No | No | No | No | No | No | No |
| 19 | de la Vega et al. (2018) | Yes | No | No | No | No | No | No | No | No | No | No |
| 20 | Ding et al. (2018) | No | Yes | No | No | No | No | No | No | No | No | No |
| 21 | Dulin et al. (2014) | No | No | Yes | No | No | No | No | No | No | No | No |
| 22 | Ferati et al. (2011) | No | No | No | No | No | No | No | No | No | No | Yes |
| 23 | Fiks et al. (2018) | Yes | No | No | No | No | No | No | No | No | No | No |
| 24 | Gray et al. (2010) | No | No | No | No | No | No | No | No | No | No | Yes |
| 25 | Groussard et al., (2018) | No | No | No | No | No | Yes | No | No | No | No | No |
| 26 | Held et al. (2017) | No | No | No | No | No | Yes | No | No | No | No | No |
| 27 | Hicks et al. (2017) | No | No | No | No | Yes | No | No | No | No | No | No |
|  |  | Systematic Review | | | | | | | | | | |
| Count | Primary Study | Azad-Khaneghah et al. (2021) | Georgsson (2020) | Inal et al. (2020) | Muro-Culebras et al. (2021) | Ng et al. (2019) | Niknejad et al. (2021) | Nouri et al. (2018) | Saeed et al. (2020) | Vera et al. (2019) | Wakefield et al. (2017) | Zapata et al. (2015) |
| 28 | Hidalgo-Mazzei et al. (2016) | No | No | No | No | Yes | No | No | No | No | No | No |
| 29 | Huguet et al. (2015) | No | No | Yes | No | No | No | No | No | No | No | No |
| 30 | Hwang et al. (2012) | No | No | No | No | No | No | No | No | No | No | Yes |
| 31 | Jimenez-Fernandez et al. (2013) | No | No | No | No | No | No | No | Yes | No | No | No |
| 32 | Jin et al. (2015) | No | No | No | No | No | No | Yes | No | No | No | No |
| 33 | Khan et al. (2012) | No | No | No | No | No | No | No | No | No | No | Yes |
| 34 | Khan et al. (2013) | No | No | No | No | No | No | No | No | No | No | Yes |
| 35 | Kho et al. (2019) | No | Yes | No | No | No | No | No | No | No | No | No |
| 36 | Klack et al. (2013) | No | No | No | No | No | No | No | Yes | No | No | No |
| 37 | Kuhn et al. (2014) | Yes | No | No | No | No | No | No | No | No | No | No |
| 38 | Kukec et al. (2011) | No | No | No | No | No | No | No | No | No | No | Yes |
| 39 | Kumar et al. (2018) | No | No | No | No | Yes | No | No | No | No | No | No |
| 40 | Latif et al. (2015) | No | No | Yes | No | No | No | No | No | No | No | No |
| 41 | Llorens et al. (2015) | No | No | No | No | No | Yes | No | No | No | No | No |
| 42 | Loy et al. (2016) | No | No | No | No | No | No | Yes | No | No | No | No |
| 43 | Martinez-Perez et al. (2015) | No | No | No | No | No | No | No | No | Yes | No | No |
| 44 | Mistler et al. (2017) | No | No | Yes | No | No | No | No | No | No | No | No |
| 45 | Morland et al. (2016) | No | No | Yes | No | No | No | No | No | No | No | No |
| 46 | Myint et al. (2016) | Yes | No | No | No | No | No | No | No | No | No | No |
| 47 | O’Connor et al. (2014) | Yes | No | No | No | No | No | No | No | No | No | No |
| 48 | Ownsworth et al. (2020) | No | No | No | No | No | Yes | No | No | No | No | No |
| 49 | Peeters et al. (2012) | No | No | No | No | No | No | No | Yes | No | No | No |
| 50 | Prada et al. (2017) | No | No | Yes | No | No | No | No | No | No | No | No |
| 51 | Price et al. (2017) | No | No | No | No | Yes | No | No | No | No | No | No |
| 52 | Ramos et al. (2017) | Yes | No | No | No | No | No | No | No | No | No | No |
| 53 | Rawstorn et al. (2018) | No | No | No | No | No | Yes | No | No | No | No | No |
| 54 | Reger et al. (2015) | No | No | No | No | Yes | No | No | No | No | No | No |
| 55 | Reynoldson et al. (2014) | No | No | No | No | No | No | No | No | No | No | Yes |
| 56 | Rizvi et al. (2016) | Yes | No | Yes | No | No | No | No | No | No | No | No |
| 57 | Rohatagi et al. (2016) | No | No | Yes | No | Yes | No | No | No | No | No | No |
|  |  | Systematic Review | | | | | | | | | | |
| Count | Primary Study | Azad-Khaneghah et al. (2021) | Georgsson (2020) | Inal et al. (2020) | Muro-Culebras et al. (2021) | Ng et al. (2019) | Niknejad et al. (2021) | Nouri et al. (2018) | Saeed et al. (2020) | Vera et al. (2019) | Wakefield et al. (2017) | Zapata et al. (2015) |
| 58 | Silva et al. (2010) | No | No | No | No | No | No | No | No | No | No | Yes |
| 59 | Stein et al. (2017) | No | Yes | No | No | No | No | No | No | No | No | No |
| 60 | Scott et al. (2015) | No | No | No | No | No | No | Yes | No | No | No | No |
| 61 | Spook et al. (2013) | Yes | No | No | No | No | No | No | No | No | No | No |
| 62 | Tay et al. (2017) | Yes | No | No | No | No | No | No | No | No | No | No |
| 63 | Welch et al. (2015) | No | Yes | No | No | No | No | No | No | No | No | No |
| 64 | Wilson et al. (2016) | Yes | No | No | No | No | No | No | No | No | No | No |
| 65 | Xiao et al. (2013) | No | No | No | No | No | No | No | No | No | No | Yes |
| 66 | Yasini et al. (2016) | No | No | No | No | No | No | Yes | No | No | No | No |

Note: no measures identified by Kien et al. (2018)

Table S5: Methods used to assess usability as identified by systematic reviews included within the present overview

|  | Georgsson (2020) | Inal et al. (2020) | Ng et al. (2019) | Niknejad et al. (2021) | Nouri et al. (2018) | Saeed et al. (2020) | Vera et al. (2019) | Zapata et al. (2015) |
| --- | --- | --- | --- | --- | --- | --- | --- | --- |
| Focus groups | Yes | Yes | NR | Yes | NR | NR | NR | Yes |
| Heuristic evaluation | Yes | NR | NR | Yes | Yes | Yes | Yes | NR |
| Interview (in-depth; semistructured) | Yes | NR | Yes | Yes | NR | Yes | Yes | Yes |
| Iterative user-centered design methodology | NR | NR | NR | NR | NR | Yes | NR | NR |
| Logs/diaries | NR | Yes | Yes | NR | Yes | NR | NR | Yes |
| (Mail) panel | Yes | Yes | NR | NR | NR | NR | NR | NR |
| Survey/Questionnaire | Yes | Yes | Yes | Yes | NR | Yes | NR | Yes |
| Think-aloud/Cognitive walk-through | Yes | Yes | NR | NR | NR | Yes | NR | Yes |
| Video/Face to Face Demonstration/Mock-Ups/direct observation | Yes  (direct observation) | Yes | NR | NR | NR | NR | NR | Yes |

NR: not reported as part of the systematic reviews included in the present overview.

Table S6: Quality assessment ratings of reviews included in the present review (=individual review level data)

|  | **AMSTAR criteria** | | | | | | | | | | | | | | | |
| --- | --- | --- | --- | --- | --- | --- | --- | --- | --- | --- | --- | --- | --- | --- | --- | --- |
|  | **1**  **(Yes, No)** | **2**  **(Yes, Partial Yes, No)** | **3**  **(Yes, No)** | **4**  **(Yes, Partial Yes, No)** | **5**  **(Yes, No)** | **6**  **(Yes, No)** | **7**  **(Yes, Partial Yes, No)** | **8**  **(Yes, Partial Yes, No)** | **9**  **(Yes, Partial Yes, No, Includes only NRSI)** | **10**  **(Yes, No)** | **11**  **(Yes, No, No meta-analysis con-ducted)** | **12**  **(Yes, No, No meta-analysis con-ducted)** | **13**  **(Yes, No)** | **14**  **(Yes, No)** | **15**  **(Yes, No, No meta-analysis con-ducted)** | **16**  **(Yes, No)** |
| Azad-Khanegha, et al. (2021) | Yes | No | Yes | Yes | Yes | No | Partial Yes | Partial Yes | No | No | No, no meta-analysis conducted | No, no meta-analysis conducted | No | Yes | No, no meta-analysis conducted | Yes |
| Georgsson (2020) | Yes | No | Yes | Partial Yes | No | No | Partial Yes | No | No | No | No, no meta-analysis conducted | No, no meta-analysis conducted | No | Yes | No, no meta-analysis conducted | Yes |
| Inal, et al. (2020) | Yes | No (as no risk of bias ax) | Yes | Partial Yes | Yes | No | No | No | No | No | No, no meta-analysis conducted | No, no meta-analysis conducted | No | Yes | No, no meta-analysis conducted | Yes |
| Kien, et al. (2018) | Yes | Yes | No | Yes | Yes | No | Partial Yes | Yes | No | No | No, no meta-analysis conducted | No, no meta-analysis conducted | No | Yes | No, no meta-analysis conducted | Yes |
| Muro-Culebras et al. (2021) | Yes | No | Yes | Yes | Yes | Yes | Partial Yes | Partial Yes | No | No | No, no meta-analysis conducted | No, no meta-analysis conducted | No | Yes | No, no meta-analysis conducted | Yes |
|  | **1**  **(Yes, No)** | **2**  **(Yes, Partial Yes, No)** | **3**  **(Yes, No)** | **4**  **(Yes, Partial Yes, No)** | **5**  **(Yes, No)** | **6**  **(Yes, No)** | **7**  **(Yes, Partial Yes, No)** | **8**  **(Yes, Partial Yes, No)** | **9**  **(Yes, Partial Yes, No, Includes only NRSI)** | **10**  **(Yes, No)** | **11**  **(Yes, No, No meta-analysis con-ducted)** | **12**  **(Yes, No, No meta-analysis con-ducted)** | **13**  **(Yes, No)** | **14**  **(Yes, No)** | **15**  **(Yes, No, No meta-analysis con-ducted)** | **16**  **(Yes, No)** |
| Ng, et al. (2019) | Yes | No | Yes | Partial Yes | Yes | Yes | No | No | No | No | No, no meta-analysis conducted | No, no meta-analysis conducted | No | Yes | No, no meta-analysis conducted | Yes |
| Niknejad, et al. (2021) | Yes | No | Yes | Partial Yes | No | Yes | No | Yes | Partial Yes | No | No, no meta-analysis conducted | No, no meta-analysis conducted | No | Yes | No, no meta-analysis conducted | Yes |
| Nouri, et al. (2018) | Yes | No | Yes | Partial Yes | Yes | No | No | Yes | No | No | No, no meta-analysis conducted | No, no meta-analysis conducted | No | Yes | No, no meta-analysis conducted | Yes |
| Saeed, et al. (2020) | Yes | No | Yes | Partial Yes | No | No | No | Partial Yes | Partial Yes | No | No, no meta-analysis conducted | No, no meta-analysis conducted | No | Yes | No, no meta-analysis conducted | Yes |
| Vera, et al. (2019) | Yes | No | Yes | Partial Yes | No | No | No | No | No | No | No, no meta-analysis conducted | No, no meta-analysis conducted | No | No | No, no meta-analysis conducted | Yes |

|  | **1**  **(Yes, No)** | **2**  **(Yes, Partial Yes, No)** | **3**  **(Yes, No)** | **4**  **(Yes, Partial Yes, No)** | **5**  **(Yes, No)** | **6**  **(Yes, No)** | **7**  **(Yes, Partial Yes, No)** | **8**  **(Yes, Partial Yes, No)** | **9**  **(Yes, Partial Yes, No, Includes only NRSI)** | **10**  **(Yes, No)** | **11**  **(Yes, No, No meta-analysis con-ducted)** | **12**  **(Yes, No, No meta-analysis con-ducted)** | **13**  **(Yes, No)** | **14**  **(Yes, No)** | **15**  **(Yes, No, No meta-analysis con-ducted)** | **16**  **(Yes, No)** |
| --- | --- | --- | --- | --- | --- | --- | --- | --- | --- | --- | --- | --- | --- | --- | --- | --- |
| Wakefield, et al. (2017) | Yes | No | Yes | Partial Yes | Yes | No | Partial Yes | Partial Yes | No | No | No, | No, no meta-analysis conducted | No | Yes | No | Yes |
| Zapata, et al. (2015) | Yes | No | Yes | Partial Yes | No | No | No | Partial Yes | No | No | No, no meta-analysis conducted | No, no meta-analysis conducted | No | Yes | No, no meta-analysis conducted | Yes |

Table S7: Citation matrix visualizing overlap of primary studies including the System Usability Scale (SUS)

|  |  | Systematic Reviews including the System Usability Scale (SUS) | | | | | | | | | | |
| --- | --- | --- | --- | --- | --- | --- | --- | --- | --- | --- | --- | --- |
|  | Study ID | Azad-Khane  ghah et al. (2021) | Georgsson (2020) | Inal et al. (2020) | Muro-Culebras et al. (2021) | Ng et al. (2019) | Niknejad et al. (2021) | Nouri et al. (2018) | Vera et al. (2019) | Wakefield et al. (2017) | Zapata et al., (2015) | Number of times included |
| Primary studies | Alnasser et al. (2018) | Yes | No | No | No | No | No | No | No | No | No | 1 |
|  | Auger et al., (2014) | No | No | Yes | No | No | No | No | No | No | No | 1 |
|  | Bauer et al. (2018) | No | No | Yes | No | Yes | No | No | No | No | No | 2 |
|  | Ben-Zeev et al (2014) | No | No | No | No | Yes | No | No | No | No | No | 1 |
|  | Bernier et al., (2018) | No | Yes | No | No | No | No | No | No | No | No | 1 |
|  | Birney et al (2016) | No | No | No | No | Yes | No | No | No | No | No | 1 |
|  | Bondaronek et al. (2018) | Yes | No | No | No | No | No | No | No | No | No | 1 |
|  | Brooke et al. (1996) | Yes | No | Yes | Yes | Yes | No | No | Yes | Yes | Yes | 7 |
|  | Brooks et al. (2015) | Yes | No | No | No | No | No | No | No | No | No | 1 |
|  | Chen et al. (2015) | No | No | No | No | No | No | Yes | No | No | No | 1 |
|  | Deady et al. (2018) | No | No | Yes | No | No | No | No | No | No | No | 1 |
|  | Dianat et al. (2014) | No | No | No | Yes | No | No | No | No | No | No | 1 |
|  | Ehrler et al. (2018) | Yes | No | No | No | No | No | No | No | No | No | 1 |
|  | Fallah et al. (2017) | Yes | No | No | No | No | No | No | No | No | No | 1 |
|  | Fritz et al. (2012) | No | No | No | No | No | No | No | No | No | Yes | 1 |
|  | Fuller-Tyszkiewicz et al. (2018) | No | No | Yes | No | No | No | No | No | No | No | 1 |
|  | Gan et al (2011) | No | No | Yes | No | No | No | Yes | No | No | No | 1 |
|  | Georgsson et al. (2016) | No | Yes | No | No | No | No | No | No | No | No | 1 |
|  | Ginsburg et al. (2015) | Yes | No | No | No | No | No | No | No | No | No | 1 |
|  | Giordanengo et al. (2019) | No | Yes | No | No | No | No | No | No | No | No | 1 |
|  | Grindrod et al. (2018) | Yes | No | No | No | No | No | No | No | No | No | 1 |
|  | Hanne Hoaas et al. (2016 | No | No | No | No | No | Yes | No | No | No | No | 1 |
|  | Hartzler et al. (2016) | No | Yes | No | No | No | No | No | No | No | No | 1 |
|  | Holzinger et al. (2011) | No | No | No | No | No | No | No | No | No | Yes | 1 |
|  | Hwang et al. (2017) | No | No | No | No | No | Yes | No | No | No | No | 1 |

|  |  | Systematic Reviews including the System Usability Scale (SUS) | | | | | | | | | | |
| --- | --- | --- | --- | --- | --- | --- | --- | --- | --- | --- | --- | --- |
|  | Study ID | Azad-Khane  ghah et al. (2021) | Georgsson (2020) | Inal et al. (2020) | Muro-Culebras et al. (2021) | Ng et al. (2019) | Niknejad et al. (2021) | Nouri et al. (2018) | Vera et al. (2019) | Wakefield et al. (2017) | Zapata et al., (2015) | Number of times included |
| Primary studies | Isakovic et al. (2016) | Yes | Yes | No | No | No | No | No | No | No | No | 2 |
|  | Ithnin et al. (2017) | Yes | No | No | No | No | No | No | No | No | No | 1 |
|  | Kizakevich et al (2018) | No | No | Yes | No | No | No | No | No | No | No | 1 |
|  | Kobak et al. (2015) | No | No | Yes | No | No | No | No | No | No | No | 1 |
|  | Levin et al (2017) | No | No | No | No | Yes | No | No | No | No | No | 1 |
|  | Martins et al (2015) | No | No | No | Yes | No | No | No | No | No | No | 1 |
|  | Marzuki et al (2018) | No | No | No | Yes | No | No | No | No | No | No | 1 |
|  | Mattson et al. (2015) | Yes | No | No | No | No | No | No | No | No | No | 1 |
|  | Nicholson et al. (2018) | No | No | Yes | No | No | No | No | No | No | No | 1 |
|  | Nitsch et al. (2016) | Yes | No | Yes | No | No | No | No | No | No | No | 2 |
|  | Op den Akker et al., (2017) | No | Yes | No | No | No | No | No | No | No | No | 1 |
|  | Plaza-Garcia et al (2017) | No | No | Yes | No | No | No | No | No | No | No | 1 |
|  | Powell et al (2016) | No | No | No | No | No | No | Yes | No | No | No | 1 |
|  | Sullivan et al. (2017) | Yes | No | No | No | No | No | No | No | No | No | 1 |
|  | Villalobos et al. (2017) | Yes | No | Yes | No | No | No | No | No | No | No | 2 |
|  | Wood et al., (2017) | No | No | Yes | No | No | No | No | No | No | No | 1 |
|  | Total studies included | 13 | 6 | 12 | 4 | 5 | 2 | 3 | See note | 1 | 3 |  |

Note: No citation details of primary studies included in systematic review by Vera et al. (2019)

**References**

Aji, M., Gordon, C., Stratton, E., Calvo, R. A., Bartlett, D., Grunstein, R., & Glozier, N. (2021). Framework for the design engineering and clinical implementation and evaluation of mHealth apps for sleep disturbance: systematic review. *JMIR*, *23*(2), e24607.

Akinola, M., Hebert, L. E., Hill, B. J., Quinn, M., Holl, J. L., Whitaker, A. K., & Gilliam, M. L. (2019). Development of a mobile app on contraceptive options for young African American and Latina women. *Health Education & Behavior*, *46*(1), 89-96.

Al Ayubi, S. U., Parmanto, B., Branch, R., & Ding, D. (2014). A persuasive and social mHealth application for physical activity: a usability and feasibility study. *JMIR mHealth and uHealth*, *2*(2), e2902.

Albanese-O'Neill, A., Schatz, D. A., Thomas, N., Bernhardt, J. M., Cook, C. L., Haller, M. J., ... & Elder, J. H. (2019). Designing online and mobile diabetes education for fathers of children with type 1 diabetes: mixed methods study. *JMIR diabetes*, *4*(3), e13724.

Aljaber, T., & Gordon, N. A. (2016). Evaluation of mobile health education applications for health professionals and patients. In *8th International Conference on e-Health (EH 2016)* (pp. 107-114).

Alnasser, A., Kyle, J., Alkhalifah, A., & Marais, D. (2018). Relationship between evidence requirements, user expectations, and actual experiences: usability evaluation of the Twazon Arabic weight loss app. *JMIR human factors*, *5*(2), e9765.

Arnhold, M., Quade, M., & Kirch, W. (2014). Mobile applications for diabetics: a systematic review and expert-based usability evaluation considering the special requirements of diabetes patients age 50 years or older. *JMIR*, *16*(4), e104.

Anderson, K., Burford, O., & Emmerton, L. (2016). App chronic disease checklist: protocol to evaluate mobile apps for chronic disease self-management. *JMIR research protocols*, *5*(4), e6194.

Atkinson, N. L. (2007). Developing a questionnaire to measure perceived attributes of eHealth innovations. *American journal of health behavior*, *31*(6), 612-621.

Auger, C., Leduc, E., Labbé, D., Guay, C., Fillion, B., Bottari, C., & Swaine, B. (2014). Mobile applications for participation at the shopping mall: Content analysis and usability for persons with physical disabilities and communication or cognitive limitations. *International Journal of Environmental Research and Public Health*, *11*(12), 12777-12794.

Azad-Khaneghah, P., Neubauer, N., Miguel Cruz, A., & Liu, L. (2021). Mobile health app usability and quality rating scales: a systematic review. *Disability and Rehabilitation: Assistive Technology*, *16*(7), 712-721.

Bauer, A. M., Iles-Shih, M., Ghomi, R. H., Rue, T., Grover, T., Kincler, N., ... & Katon, W. J. (2018). Acceptability of mHealth augmentation of Collaborative Care: A mixed methods pilot study. *General hospital psychiatry*, *51*, 22-29.

Baumel, A., Birnbaum, M. L., & Sucala, M. (2017). A systematic review and taxonomy of published quality criteria related to the evaluation of user-facing eHealth programs. *Journal of medical systems*, *41*, 1-7.

Ben-Zeev, D., Brenner, C. J., Begale, M., Duffecy, J., Mohr, D. C., & Mueser, K. T. (2014). Feasibility, acceptability, and preliminary efficacy of a smartphone intervention for schizophrenia. *Schizophrenia bulletin*, *40*(6), 1244-1253.

Ben-Zeev, D., Wang, R., Abdullah, S., Brian, R., Scherer, E. A., Mistler, L. A., ... & Choudhury, T. (2016). Mobile behavioral sensing for outpatients and inpatients with schizophrenia. *Psychiatric services*, *67*(5), 558-561.

Ben-Zeev, D., Brian, R. M., Aschbrenner, K. A., Jonathan, G., & Steingard, S. (2018). Video-based mobile health interventions for people with schizophrenia: Bringing the “pocket therapist” to life. *Psychiatric rehabilitation journal*, *41*(1), 39.

Bernier, A., Fedele, D., Guo, Y., Chavez, S., Smith, M. D., Warnick, J., ... & Modave, F. (2018). New-onset diabetes educator to educate children and their caregivers about diabetes at the time of diagnosis: usability study. *JMIR diabetes*, *3*(2), e9202.

Binyamin, S. S., & Zafar, B. A. (2021). Proposing a mobile apps acceptance model for users in the health area: A systematic literature review and meta-analysis. *Health Informatics Journal*, *27*(1), 1460458220976737.

Birney, A. J., Gunn, R., Russell, J. K., & Ary, D. V. (2016). MoodHacker mobile web app with email for adults to self-manage mild-to-moderate depression: randomized controlled trial. *JMIR mHealth and uHealth*, *4*(1), e4231.

Boisseau, C. L., Schwartzman, C. M., Lawton, J., & Mancebo, M. C. (2017). App-guided exposure and response prevention for obsessive compulsive disorder: an open pilot trial. *Cognitive Behaviour Therapy*, *46*(6), 447-458.

Boman, I. L., & Bartfai, A. (2015). The first step in using a robot in brain injury rehabilitation: patients’ and health-care professionals’ perspective. *Disability and Rehabilitation: Assistive Technology*, *10*(5), 365-370.

Bondaronek, P., Alkhaldi, G., Slee, A., Hamilton, F. L., & Murray, E. (2018). Quality of publicly available physical activity apps: review and content analysis. *JMIR mHealth and uHealth*, *6*(3), e9069.

Bonten, T. N., Rauwerdink, A., Wyatt, J. C., Kasteleyn, M. J., Witkamp, L., Riper, H., ... & EHealth Evaluation Research Group. (2020). Online guide for electronic health evaluation approaches: systematic scoping review and concept mapping study. *JMIR*, *22*(8), e17774.

Brooke, J. (1996). SUS: A quick and dirty usability scale. *Usability Evaluation in Industry*.

Brooks, G. C., Vittinghoff, E., Iyer, S., Tandon, D., Kuhar, P., Madsen, K. A., ... & Olgin, J. E. (2015). Accuracy and usability of a self-administered 6-minute walk test smartphone application. *Circulation: Heart Failure*, *8*(5), 905-913.

Chen, J., Cade, J. E., & Allman-Farinelli, M. (2015). The most popular smartphone apps for weight loss: a quality assessment. *JMIR mHealth and uHealth*, *3*(4), e4334.

Chenchen, G., Zhou, L., Liu, Z., Wang, H., & Bowers, B. (2017). Mobile application for diabetes self-management in China: Do they fit for older adults? *International journal of medical informatics*, *101*, 68-74. Georgsson, M. (2020). A review of usability methods used in the evaluation of mobile health applications for diabetes. *pHealth 2020*, 228-233.

Cho, M. J., Sim, J. L., & Hwang, S. Y. (2014). Development of smartphone educational application for patients with coronary artery disease. *Healthcare informatics research*, *20*(2), 117-124.

Connelly, K., Stein, K. F., Chaudhry, B., & Trabold, N. (2016). Development of an ecological momentary assessment mobile app for a low-literacy, Mexican American population to collect disordered eating behaviors. *JMIR public health and surveillance*, *2*(2), e5511.

Cruz Zapata, B., Hernandez Ninirola, A., Idri, A., Fernández-Alemán, J. L., & Toval, A. (2014). Mobile PHRs compliance with Android and iOS usability guidelines. Journal of medical systems, 38, 1-16.

de la Torre Díez, I., Garcia-Zapirain, B., López-Coronado, M., Rodrigues, J. J., & del Pozo Vegas, C. (2017). A new mHealth app for monitoring and awareness of healthy eating: development and user evaluation by Spanish users. *Journal of medical systems*, *41*, 1-7.

de la Vega, R., Roset, R., Galán, S., & Miró, J. (2018). Fibroline: A mobile app for improving the quality of life of young people with fibromyalgia. *Journal of Health Psychology*, *23*(1), 67-78.

Deady, M., Johnston, D., Milne, D., Glozier, N., Peters, D., Calvo, R., & Harvey, S. Preliminary effectiveness of a smartphone app to reduce depressive symptoms in the workplace: feasibility and acceptability study. JMIR Mhealth Uhealth. 2018 Dec 04; 6 (12): e11661. 10.2196/11661.

Dianat, I., Ghanbari, Z., & AsghariJafarabadi, M. (2014). Psychometric properties of the Persian language version of the system usability scale. *Health promotion perspectives*, *4*(1), 82.

Ding, H., Fatehi, F., Russell, A. W., Karunanithi, M., Menon, A., Bird, D., & Gray, L. C. (2018). User experience of an innovative mobile health program to assist in insulin dose adjustment: outcomes of a proof-of-concept trial. *Telemedicine and e-Health*, *24*(7), 536-543.

Dulin, P. L., Gonzalez, V. M., & Campbell, K. (2014). Results of a pilot test of a self-administered smartphone-based treatment system for alcohol use disorders: usability and early outcomes. *Substance abuse*, *35*(2), 168-175.

Ehrler, F., Weinhold, T., Joe, J., Lovis, C., & Blondon, K. (2018). A mobile app (BEDSide Mobility) to support nurses’ tasks at the patient's bedside: usability study. *JMIR mHealth and uHealth*, *6*(3), e9079.

Fallah, M., & Yasini, M. (2017). A medication reminder mobile app: does it work for different age ranges. In *Informatics for Health: Connected Citizen-Led Wellness and Population Health* (pp. 68-72). IOS press.

Ferati, M., Mannheimer, S., & Bolchini, D. (2011, October). Usability evaluation of acoustic interfaces for the blind. In *Proceedings of the 29th ACM international conference on Design of communication* (pp. 9-16).

Fiks, A. G., Fleisher, L., Berrigan, L., Sykes, E., Mayne, S. L., Gruver, R., ... & McMahon, P. (2018). Usability, acceptability, and impact of a pediatric teledermatology mobile health application. *Telemedicine and e-Health*, *24*(3), 236-245.

Fritz, F., Balhorn, S., Riek, M., Breil, B., & Dugas, M. (2012). Qualitative and quantitative evaluation of EHR-integrated mobile patient questionnaires regarding usability and cost-efficiency. *International Journal of Medical Informatics*, *81*(5), 303-313.

Fuller-Tyszkiewicz, M., Richardson, B., Klein, B., Skouteris, H., Christensen, H., Austin, D., ... & Ware, A. (2018). A mobile app–based intervention for depression: End-user and expert usability testing study. *JMIR mental health*, *5*(3), e9445.

Gay, V., Leijdekkers, P., & Barin, E. (2010, July). Feasibility trial of a novel mobile cardiac rehabilitation application. In *The 12th IEEE International Conference on e-Health Networking, Applications and Services* (pp. 86-92). IEEE.

Georgsson, M. (2020). A review of usability methods used in the evaluation of mobile health applications for diabetes. *pHealth 2020*, 228-233.

Georgsson, M., Staggers, N., & Weir, C. (2016). A modified user-oriented heuristic evaluation of a mobile health system for diabetes self-management support. *CIN: Computers, Informatics, Nursing*, *34*(2), 77-84.

Ginsburg, A. S., Delarosa, J., Brunette, W., Levari, S., Sundt, M., Larson, C., ... & Anderson, R. (2015). mPneumonia: development of an innovative mHealth application for diagnosing and treating childhood pneumonia and other childhood illnesses in low-resource settings. *PloS one*, *10*(10), e0139625.

Giordanengo, A., Årsand, E., Woldaregay, A. Z., Bradway, M., Grottland, A., Hartvigsen, G., ... & Hansen, A. H. (2019). Design and prestudy assessment of a dashboard for presenting self-collected health data of patients with diabetes to clinicians: Iterative approach and qualitative case study. *JMIR diabetes*, *4*(3), e14002.

Grindrod, K., Khan, H., Hengartner, U., Ong, S., Logan, A. G., Vogel, D., ... & Yang, J. (2018). Evaluating authentication options for mobile health applications in younger and older adults. *PloS one*, *13*(1), e0189048.

Grundy, Q. H., Wang, Z., & Bero, L. A. (2016). Challenges in assessing mobile health app quality: a systematic review of prevalent and innovative methods. *American journal of preventive medicine*, *51*(6), 1051-1059.

Hartzler, A. L., Venkatakrishnan, A., Mohan, S., Silva, M., Lozano, P., Ralston, J. D., ... & Pirolli, P. (2016, August). Acceptability of a team-based mobile health (mHealth) application for lifestyle self-management in individuals with chronic illnesses. In *2016 38th Annual International Conference of the IEEE Engineering in Medicine and Biology Society (EMBC)* (pp. 3277-3281). IEEE.

Held, J. P., Ferrer, B., Mainetti, R., Steblin, A., Hertler, B., Moreno-Conde, A., ... & Borghese, N. A. (2017). Autonomous rehabilitation at stroke patients home for balance and gait: safety, usability and compliance of a virtual reality system. *European journal of physical and rehabilitation medicine*.

Hicks, T. A., Thomas, S. P., Wilson, S. M., Calhoun, P. S., Kuhn, E. R., & Beckham, J. C. (2017). A preliminary investigation of a relapse prevention mobile application to maintain smoking abstinence among individuals with posttraumatic stress disorder. *Journal of dual diagnosis*, *13*(1), 15-20.

Hidalgo-Mazzei, D., Mateu, A., Reinares, M., Murru, A., del Mar Bonnín, C., Varo, C., ... & Colom, F. (2016). Psychoeducation in bipolar disorder with a SIMPLe smartphone application: feasibility, acceptability and satisfaction. *Journal of Affective Disorders*, *200*, 58-66.

Hoaas, H., Andreassen, H. K., Lien, L. A., Hjalmarsen, A., & Zanaboni, P. (2016). Adherence and factors affecting satisfaction in long-term telerehabilitation for patients with chronic obstructive pulmonary disease: a mixed methods study. *BMC medical informatics and decision making*, *16*, 1-14.

Holzinger, A., Kosec, P., Schwantzer, G., Debevc, M., Hofmann-Wellenhof, R., & Frühauf, J. (2011). Design and development of a mobile computer application to reengineer workflows in the hospital and the methodology to evaluate its effectiveness. *Journal of biomedical informatics*, *44*(6), 968-977.

Hosseiniravandi, M., Kahlaee, A. H., Karim, H., Ghamkhar, L., & Safdari, R. (2020). Home-based telerehabilitation software systems for remote supervising: a systematic review. *International journal of technology assessment in health care*, *36*(2), 113-125.

Huguet, A., McGrath, P. J., Wheaton, M., Mackinnon, S. P., Rozario, S., Tougas, M. E., ... & MacLean, C. (2015). Testing the feasibility and psychometric properties of a mobile diary (myWHI) in adolescents and young adults with headaches. *JMIR mHealth and uHealth*, *3*(2), e3879.

Hwang, Y., Shin, D., Yang, C. Y., Lee, S. Y., Kim, J., Kong, B., ... & Chung, M. (2012). Developing a voice user interface with improved usability for people with dysarthria. In *Computers Helping People with Special Needs: 13th International Conference, ICCHP 2012, Linz, Austria, July 11-13, 2012, Proceedings, Part II 13* (pp. 117-124). Springer Berlin Heidelberg.

Inal, Y., Wake, J. D., Guribye, F., & Nordgreen, T. (2020). Usability evaluations of mobile mental health technologies: systematic review. *Journal of medical Internet research*, *22*(1), e15337.

Isaković, M., Sedlar, U., Volk, M., & Bešter, J. (2016). Usability pitfalls of diabetes mHealth apps for the elderly. *Journal of diabetes research*, *2016*(1), 1604609.

Ithnin, M., Rani, M. D. M., Abd Latif, Z., Kani, P., Syaiful, A., Aripin, K. N. N., & Mohd, T. A. M. T. (2017). Mobile app design, development, and publication for adverse drug reaction assessments of causality, severity, and preventability. *JMIR mHealth and uHealth*, *5*(5), e6261.

Jiménez-Fernández, S., De Toledo, P., & Del Pozo, F. (2013). Usability and interoperability in wireless sensor networks for patient telemonitoring in chronic disease management. *IEEE Transactions on Biomedical Engineering*, *60*(12), 3331-3339.

Jin, M., & Kim, J. (2015). Development and evaluation of an evaluation tool for healthcare smartphone applications. *Telemedicine and e-Health*, *21*(10), 831-837.

Khan, D. U., Ananthanarayan, S., Le, A. T., Schaefbauer, C. L., & Siek, K. A. (2012, May). Designing mobile snack application for low socioeconomic status families. In *2012 6th international conference on pervasive computing technologies for healthcare (pervasivehealth) and workshops* (pp. 57-64). IEEE.

Khan, S., Tahir, M. N., & Raza, A. (2013, December). Usability issues for smartphone users with special needs—Autism. In *2013 international conference on open source systems and technologies* (pp. 107-113). IEEE.

Kho, S. E. S., Lim, S. G., Hoi, W. H., Ng, P. L., Tan, L., & Kowitlawakul, Y. (2019). The development of a diabetes application for patients with poorly controlled type 2 diabetes mellitus. *CIN: Computers, Informatics, Nursing*, *37*(2), 99-106.

Kien, C., Schultes, M. T., Szelag, M., Schoberberger, R., & Gartlehner, G. (2018). German language questionnaires for assessing implementation constructs and outcomes of psychosocial and health-related interventions: a systematic review. *Implementation Science*, *13*, 1-16.

Kizakevich, P. N., Eckhoff, R., Brown, J., Tueller, S. J., Weimer, B., Bell, S., ... & King, L. A. (2018). PHIT for duty, a mobile application for stress reduction, sleep improvement, and alcohol moderation. *Military medicine*, *183*(suppl_1), 353-363.

Klack, L., Ziefle, M., Wilkowska, W., & Kluge, J. (2013). Telemedical versus conventional heart patient monitoring: a survey study with German physicians. *International journal of technology assessment in health care*, *29*(4), 378-383.

Kobak, K. A., Mundt, J. C., & Kennard, B. (2015). Integrating technology into cognitive behavior therapy for adolescent depression: a pilot study. *Annals of general psychiatry*, *14*, 1-10.

Kuhn, E., Greene, C., Hoffman, J., Nguyen, T., Wald, L., Schmidt, J., ... & Ruzek, J. (2014). Preliminary evaluation of PTSD Coach, a smartphone app for post-traumatic stress symptoms. *Military medicine*, *179*(1), 12-18.

Kukec, M., Ljubic, S., & Glavinic, V. (2011). Need for usability and wish for mobility: case study of client end applications for primary healthcare providers in Croatia. In Information Quality in e-Health: 7th Conference of the Workgroup Human-Computer Interaction and Usability Engineering of the Austrian Computer Society, USAB 2011, Graz, Austria, November 25-26, 2011. Proceedings 7 (pp. 171-190). Springer Berlin Heidelberg.

Kumar, S., Southard, P. B., & White, M. (2016). Telemedicine: determining" critical to quality" characteristics for a healthcare service system design based on a survey of physical rehabilitation providers. *IEEE Engineering Management Review*, *44*(2), 41-55.

Kumar, D., Tully, L. M., Iosif, A. M., Zakskorn, L. N., Nye, K. E., Zia, A., & Niendam, T. A. (2018). A mobile health platform for clinical monitoring in early psychosis: implementation in community-based outpatient early psychosis care. *JMIR mental health*, *5*(1), e8551.

Larbi, D., Randine, P., Årsand, E., Antypas, K., Bradway, M., & Gabarron, E. (2020). Methods and evaluation criteria for apps and digital interventions for diabetes self-management: systematic review. *JMIR*, *22*(7), e18480.

Latif, S., Tariq, R., Tariq, S., & Latif, R. (2015). Designing an assistive learning aid for writing acquisition: a challenge for children with dyslexia. In *Assistive Technology* (pp. 180-188). IOS Press.

Lee, S., Lee, Y., Lee, S., Islam, S. M. S., & Kim, S. Y. (2019). Toward developing a standardized core set of outcome measures in mobile health interventions for tuberculosis management: systematic review. *JMIR mHealth and uHealth*, *7*(2), e12385.

Levin, M. E., Haeger, J., Pierce, B., & Cruz, R. A. (2017). Evaluating an adjunctive mobile app to enhance psychological flexibility in acceptance and commitment therapy. *Behavior Modification*, *41*(6), 846-867.

Loy, J. S., Ali, E. E., & Yap, K. Y. L. (2016). Quality assessment of medical apps that target medication-related problems. *Journal of managed care & specialty pharmacy*, *22*(10), 1124-1140.

Martins, A. I., Rosa, A. F., Queirós, A., Silva, A., & Rocha, N. P. (2015). European Portuguese validation of the system usability scale (SUS). *Procedia computer science*, *67*, 293-300.

Martínez-Pérez, B., de la Torre-Díez, I., & López-Coronado, M. (2015). Experiences and results of applying tools for assessing the quality of a mHealth app named Heartkeeper. *Journal of medical systems*, *39*, 1-6.

Marzuki, M. F. M., Yaacob, N. A., & Yaacob, N. M. (2018). Translation, cross-cultural adaptation, and validation of the Malay version of the system usability scale questionnaire for the assessment of mobile apps. *JMIR human factors*, *5*(2), e10308.

Mattson, D. C. (2015). Usability assessment of a mobile app for art therapy. *The Arts in Psychotherapy*.

Mistler, L. A., Ben-Zeev, D., Carpenter-Song, E., Brunette, M. F., & Friedman, M. J. (2017). Mobile mindfulness intervention on an acute psychiatric unit: feasibility and acceptability study. *JMIR Mental Health*, *4*(3), e7717.

Morland, L. A., Niehaus, J., Taft, C., Marx, B. P., Menez, U., & Mackintosh, M. A. (2016). Using a mobile application in the management of anger problems among veterans: a pilot study. *Military medicine*, *181*(9), 990-995.

Moshi, M. R., Tooher, R., & Merlin, T. (2018). Suitability of current evaluation frameworks for use in the health technology assessment of mobile medical applications: a systematic review. *International Journal of Technology Assessment in Health Care*, *34*(5), 464-475.

Muntaner, A., Vidal-Conti, J., & Palou, P. (2016). Increasing physical activity through mobile device interventions: A systematic review. *Health informatics journal*, *22*(3), 451-469.

Muro-Culebras, A., Escriche-Escuder, A., Martin-Martin, J., Roldán-Jiménez, C., De-Torres, I., Ruiz-Muñoz, M., ... & Cuesta-Vargas, A. I. (2021). Tools for evaluating the content, efficacy, and usability of mobile health apps according to the consensus-based standards for the selection of health measurement instruments: systematic review. *JMIR mHealth and uHealth*, *9*(12), e15433.

Myint, M., Adam, A., Herath, S., & Smith, G. (2016). Mobile phone applications in management of enuresis: The good, the bad, and the unreliable! *Journal of pediatric urology*, *12*(2), 112-e1.

Ng, M. M., Firth, J., Minen, M., & Torous, J. (2019). User engagement in mental health apps: a review of measurement, reporting, and validity. *Psychiatric Services*, *70*(7), 538-544.

Nicholson, J., Wright, S. M., Carlisle, A. M., Sweeney, M. A., & McHugo, G. J. (2018). The WorkingWell mobile phone app for individuals with serious mental illnesses: proof-of-concept, mixed-methods feasibility study. *JMIR mental health*, *5*(4), e11383.

Niknejad, N., Ismail, W., Bahari, M., & Nazari, B. (2021). Understanding telerehabilitation technology to evaluate stakeholders’ adoption of telerehabilitation services: a systematic literature review and directions for further research. *Archives of Physical Medicine and Rehabilitation*, *102*(7), 1390-1403.

Nitsch, M., Dimopoulos, C. N., Flaschberger, E., Saffran, K., Kruger, J. F., Garlock, L., ... & Jones, M. (2016). A guided online and mobile self-help program for individuals with eating disorders: an iterative engagement and usability study. *Journal of medical Internet research*, *18*(1), e7.

Nouri, R., R Niakan Kalhori, S., Ghazisaeedi, M., Marchand, G., & Yasini, M. (2018). Criteria for assessing the quality of mHealth apps: a systematic review. *Journal of the American Medical Informatics Association*, *25*(8), 1089-1098.

O'Connor, E., Farrow, M., & Hatherly, C. (2014). Randomized comparison of mobile and web-tools to provide dementia risk reduction education: use, engagement and participant satisfaction. *JMIR Mental Health*, *1*(1), e3654.

Op den Akker, H.J.A., Klaassen, R., Bul, K., Kato, P. M., van der Burg, G. J., & Di Bitonto, P. (2017, May). Let them play: Experiences in the wild with a gamification and coaching system for young diabetes patients. In *Proceedings of the 11th EAI international conference on pervasive computing technologies for healthcare* (pp. 409-418).

Ownsworth, T., Theodoros, D., Cahill, L., Vaezipour, A., Quinn, R., Kendall, M., ... & Lucas, K. (2020). Perceived usability and acceptability of videoconferencing for delivering community-based rehabilitation to individuals with acquired brain injury: a qualitative investigation. *Journal of the International Neuropsychological Society*, *26*(1), 47-57.

Ovčjak, B., Heričko, M., & Polančič, G. (2015). Factors impacting the acceptance of mobile data services–A systematic literature review. *Computers in human behavior*, *53*, 24-47.

Peeters, J. M., de Veer, A. J., van der Hoek, L., & Francke, A. L. (2012). Factors influencing the adoption of home telecare by elderly or chronically ill people: a national survey. *Journal of clinical nursing*, *21*(21-22), 3183-3193.

Plaza-García, I. P., Sánchez, C. M., Espílez, Á. S., García-Magariño, I., Guillén, G. A., & García-Campayo, J. (2017). Development and initial evaluation of a mobile application to help with mindfulness training and practice. *International journal of medical informatics*, *105*, 59-67.

Powell, A. C., Torous, J., Chan, S., Raynor, G. S., Shwarts, E., Shanahan, M., & Landman, A. B. (2016). Interrater reliability of mHealth app rating measures: analysis of top depression and smoking cessation apps. *JMIR mHealth and uHealth*, *4*(1), e5176.

Prada, P., Zamberg, I., Bouillault, G., Jimenez, N., Zimmermann, J., Hasler, R., ... & Perroud, N. (2017). EMOTEO: a smartphone application for monitoring and reducing aversive tension in borderline personality disorder patients, a pilot study. *Perspectives in psychiatric care*, *53*(4), 289-298.

Price, M., van Stolk-Cooke, K., Ward, H. L., O’Keefe, M., Gratton, J., Skalka, C., & Freeman, K. (2017). Tracking post-trauma psychopathology using mobile applications: a usability study. *Journal of Technology in Behavioral Science*, *2*, 41-48.

Radbron, E., Wilson, V., McCance, T., & Middleton, R. (2019). The Use of Data Collected from mHealth Apps to Inform Evidence‐Based Quality Improvement: An Integrative Review. *Worldviews on Evidence‐Based Nursing*, *16*(1), 70-77.

Ramos, S. R. (2017). User-centered design, experience, and usability of an electronic consent user interface to facilitate informed decision-making in an HIV clinic. *CIN: Computers, Informatics, Nursing*, *35*(11), 556-564.

Rawstorn, J. C., Gant, N., Rolleston, A., Whittaker, R., Stewart, R., Benatar, J., ... & Maddison, R. (2018). End users want alternative intervention delivery models: usability and acceptability of the REMOTE-CR exercise-based cardiac telerehabilitation program. *Archives of physical medicine and rehabilitation*, *99*(11), 2373-2377.

Reger, G. M., Skopp, N. A., Edwards-Stewart, A., & Lemus, E. L. (2015). Comparison of prolonged exposure (PE) coach to treatment as usual: A case series with two active duty soldiers. *Military Psychology*, *27*(5), 287-296.

Reynoldson, C., Stones, C., Allsop, M., Gardner, P., Bennett, M. I., Closs, S. J., ... & Knapp, P. (2014). Assessing the quality and usability of smartphone apps for pain self-management. *Pain medicine*, *15*(6), 898-909.

Rizvi, S. L., Hughes, C. D., & Thomas, M. C. (2016). The DBT Coach mobile application as an adjunct to treatment for suicidal and self-injuring individuals with borderline personality disorder: A preliminary evaluation and challenges to client utilization. *Psychological services*, *13*(4), 380.

Rohatagi, S., Profit, D., Hatch, A., Zhao, C., Docherty, J. P., & Peters-Strickland, T. S. (2016). Optimization of a digital medicine system in psychiatry. *The Journal of Clinical Psychiatry*, *77*(9), 13486.

Saeed, N., Manzoor, M., & Khosravi, P. (2020). An exploration of usability issues in telecare monitoring systems and possible solutions: a systematic literature review. *Disability and Rehabilitation: Assistive Technology*, *15*(3), 271-281.

Scott, K., Richards, D., & Adhikari, R. (2015). A review and comparative analysis of security risks and safety measures of mobile health apps. *Australasian Journal of Information Systems*, *19*, 1-18.

Spook, J. E., Paulussen, T., Kok, G., & Van Empelen, P. (2013). Monitoring dietary intake and physical activity electronically: feasibility, usability, and ecological validity of a mobile-based Ecological Momentary Assessment tool. *Journal of medical Internet research*, *15*(9), e214.

Stein, N., & Brooks, K. (2017). A fully automated conversational artificial intelligence for weight loss: longitudinal observational study among overweight and obese adults. *JMIR diabetes*, *2*(2), e8590.

Sullivan, P. S., Driggers, R., Stekler, J. D., Siegler, A., Goldenberg, T., McDougal, S. J., ... & Stephenson, R. (2017). Usability and acceptability of a mobile comprehensive HIV prevention app for men who have sex with men: a pilot study. *JMIR mHealth and uHealth*, *5*(3), e7199. Tay, I., Garland, S., Gorelik, A., & Wark, J. D. (2017). Development and testing of a mobile phone app for self-monitoring of calcium intake in young women. *JMIR mHealth and uHealth*, *5*(3), e5717.

Vera, F., Noël, R., & Taramasco, C. (2019). Standards, processes and instruments for assessing usability of health mobile apps: a systematic literature review. *MedInfo*, 1797-1798.

Villalobos, O., Lynch, S., DeBlieck, C., & Summers, L. (2017). Utilization of a mobile app to assess psychiatric patients with limited English proficiency. *Hispanic Journal of Behavioral Sciences*, *39*(3), 369-380.

Wakefield, B. J., Turvey, C. L., Nazi, K. M., Holman, J. E., Hogan, T. P., Shimada, S. L., & Kennedy, D. R. (2017). Psychometric properties of patient-facing eHealth evaluation measures: systematic review and analysis. *Journal of medical Internet research*, *19*(10), e346.

Welch, G., Balder, A., & Zagarins, S. (2015). Telehealth program for type 2 diabetes: usability, satisfaction, and clinical usefulness in an urban community health center. *Telemedicine and e-Health*, *21*(5), 395-403.

Wilson, K., Atkinson, K. M., Westeinde, J., Bell, C., Marty, K., Fergusson, D., ... & Bettinger, J. A. (2016). An evaluation of the feasibility and usability of a proof of concept mobile app for adverse event reporting post influenza vaccination. *Human vaccines & immunotherapeutics*, *12*(7), 1738-1748.

Wood, A. E., Prins, A., Bush, N. E., Hsia, J. F., Bourn, L. E., Earley, M. D., ... & Ruzek, J. (2017). Reduction of burnout in mental health care providers using the provider resilience mobile application. *Community mental health journal*, *53*, 452-459.

Xiao, B., Asghar, M. Z., Jämsä, T., & Pulii, P. (2013, November). " Canderoid": A mobile system to remotely monitor travelling status of the elderly with dementia. In *2013 International Joint Conference on Awareness Science and Technology & Ubi-Media Computing (iCAST 2013 & UMEDIA 2013)* (pp. 648-654). IEEE.

Yasini, M., Beranger, J., Desmarais, P., Perez, L., & Marchand, G. (2016). mHealth quality: a process to seal the qualified mobile health apps. In *Exploring complexity in health: An interdisciplinary systems approach* (pp. 205-209). IOS Press.

Yoshida, Y., Patil, S. J., Brownson, R. C., Boren, S. A., Kim, M., Dobson, R., ... & Simoes, E. J. (2020). Using the RE-AIM framework to evaluate internal and external validity of mobile phone–based interventions in diabetes self-management education and support. *Journal of the American Medical Informatics Association*, *27*(6), 946-956.

Zapata, B. C., Fernández-Alemán, J. L., Idri, A., & Toval, A. (2015). Empirical studies on usability of mHealth apps: a systematic literature review. *Journal of medical systems*, *39*, 1-19.

Zhou, L., Bao, J., & Parmanto, B. (2017). Systematic review protocol to assess the effectiveness of usability questionnaires in mhealth app studies. JMIR research protocols, 6(8), e7826.

Zischke, C., Simas, V., Hing, W., Milne, N., Spittle, A., & Pope, R. (2021). The utility of physiotherapy assessments delivered by telehealth: A systematic review. *Journal of global health*, *11*.
